# Combined Acute Ozone and Water Stress Alters the Quantitative Relationships between O_3_ Uptake, Photosynthetic Characteristics and Volatile Emissions in *Brassica nigra*

**DOI:** 10.3390/molecules26113114

**Published:** 2021-05-23

**Authors:** Kaia Kask, Eve Kaurilind, Eero Talts, Astrid Kännaste, Ülo Niinemets

**Affiliations:** 1 Chair of Crop Science and Plant Biology, Institute of Agricultural and Environmental Sciences, Estonian University of Life Sciences, Kreutzwaldi 1, 51006 Tartu, Estonia; eve.kaurilind@gmail.com (E.K.); eero.talts@emu.ee (E.T.); astrid.kannaste@emu.ee (A.K.); ylo.niinemets@emu.ee (Ü.N.); 2 Estonian Academy of Sciences, Kohtu 6, 10130 Tallinn, Estonia

**Keywords:** Brassicales, drought, lipoxygenase pathway volatiles, oxidative stress, O_3_, quantitative stress responses, volatile glucosinolates, volatile organic compounds

## Abstract

Ozone (O_3_) entry into plant leaves depends on atmospheric O_3_ concentration, exposure time and openness of stomata. O_3_ negatively impacts photosynthesis rate (*A*) and might induce the release of reactive volatile organic compounds (VOCs) that can quench O_3_, and thereby partly ameliorate O_3_ stress. Water stress reduces stomatal conductance (*g*_s_) and O_3_ uptake and can affect VOC release and O_3_ quenching by VOC, but the interactive effects of O_3_ exposure and water stress, as possibly mediated by VOC, are poorly understood. Well-watered (WW) and water-stressed (WS) *Brassica nigra* plants were exposed to 250 and 550 ppb O_3_ for 1 h, and O_3_ uptake rates, photosynthetic characteristics and VOC emissions were measured through 22 h recovery. The highest O_3_ uptake was observed in WW plants exposed to 550 ppb O_3_ with the greatest reduction and poorest recovery of *g*_s_ and *A*, and elicitation of lipoxygenase (LOX) pathway volatiles 10 min–1.5 h after exposure indicating cellular damage. Ozone uptake was similar in 250 ppb WW and 550 ppb WS plants and, in both treatments, O_3_-dependent reduction in photosynthetic characteristics was moderate and fully reversible, and VOC emissions were little affected. Water stress alone did not affect the total amount and composition of VOC emissions. The results indicate that drought ameliorated O_3_ stress by reducing O_3_ uptake through stomatal closure and the two stresses operated in an antagonistic manner in *B. nigra*.

## 1. Introduction

Tropospheric O_3_ is a phytotoxin and considered as one of the most damaging greenhouse gases; it is mainly formed via photochemical reactions involving nitrogen oxides (NO_x_) and volatile organic compounds (VOCs) [1,2,3]. According to recent studies, tropospheric O_3_ concentration is beginning to stabilize [4,5,6]; nevertheless, the current O_3_ levels reduce plant photosynthesis and growth rates and decrease potential agricultural productivity in many areas of the world [7,8,9]. Average O_3_ concentrations vary between 14.5 and 70.1 ppb during the growing season in the forests of Europe [10], but during heat waves O_3_ concentrations can be even higher [11,12].

The impact of O_3_ is often studied using short-term acute treatments when plants are exposed to high O_3_ for several minutes to some hours, and resultant visible damage symptoms on leaves and modifications in photosynthetic activity are monitored [13,14,15]. O_3_ enters in the leaves through stomata and the O_3_ exposure inhibits stomatal conductance to water vapor (*g*_s_) [16] and leads to reductions in net assimilation rate (*A*), whereas plants may or may not recover depending on the O_3_ dose [17]. O_3_ also activates the production of reactive oxygen species (ROS) [18,19], which in turn activate various defense reactions related to non-volatile and/or volatile metabolites depending on the plant species and genotype [20,21,22,23,24]. Characteristically, O_3_ exposure leads to emissions of methanol and lipoxygenase (LOX) pathway volatiles due to damaged cell walls and cell and organelle membranes [23,25] and might also result in emissions of the stress hormone methyl salicylate [20,23,26].

Next to O_3_, drought stress is estimated to have become more widespread and severe due to climate change [27,28]. It is relatively well known how reduced water availability impacts plant photosynthetic characteristics, including reductions of the assimilation rate due to stomatal closure for mild to moderately severe water deficits, and stomatal and non-stomatal reductions of the assimilation rate for more severe and sustained water stress [29,30,31,32]. There is much less information on how plant constitutive VOC emission is affected by drought. Mild to moderate water stress can enhance and severe stress curb VOC emissions due to substrate limitations resulting from reduced photosynthetic carbon input [33,34,35,36]. However, constitutive VOC emission levels strongly vary among species [37,38,39], and the substrate limitations might not necessarily limit the emissions in relatively low volatile emitters. Furthermore, drought stress differs from other abiotic stresses, such as heat, in that it seldom results in induction of stress volatiles [40,41] or only leads to a minor elicitation of stress volatiles [26]. Although drought itself does not necessarily lead to elicitation of stress volatiles, it could prime the plants to subsequent stresses, implying modified responses of photosynthetic and volatile emission characteristics upon exposure to a different stress such as ozone stress [26,40,42,43]. Yet, only a few studies have investigated the impact of combined drought and ozone stresses and simultaneously monitored both photosynthetic and VOC emission characteristics [26]. Although both drought and O_3_ reduce *g*_s_, O_3_ can cause stomatal sluggishness, i.e., reduce the responsiveness of stomata to environmental clues such as light and humidity, disturbing the effective control of transpiration [44] and making the plants more vulnerable to the following stresses [45]. On the other hand, drought that precedes O_3_ stress can reduce stomatal ozone uptake and thereby reduce O_3_-dependent damage [36,46], but whether the impact of drought on plant O_3_ response is only due to reduced O_3_ uptake or whether there is a significant interactive effect among drought and O_3_ on photosynthesis and volatile emissions, e.g., due to drought priming, is not known.

Brassicaceae constitute an important plant family that includes many agriculturally important species and their wild relatives. Species from this family are typically low-level constitutive volatile emitters [47,48], but they include a myrosinase/glucosinolate defense system, constituting of separately stored myrosinase enzymes and non-volatile glucosinolates [49,50,51]. Upon cellular damage, myrosinase comes into contact with glucosinolates, leading to synthesis of toxic volatile glucosinolate breakdown products that result in a characteristic blend of volatiles [52,53]. Volatile glucosinolate emissions are usually associated with herbivory, but severe abiotic stress that results in cellular damage, such as heat stress, can also lead to emissions of glucosinolate breakdown products [47]. So far, the available evidence of the O_3_ effects on glucosinolates is inconclusive. In O_3_-stressed *B. campestris* L. ssp. *chinensis* [54] and *B. napus* [55], the content of different glucosinolates was differently affected. In another study, O_3_ levels of 70, 80 and 120 ppb had no significant effect on VOCs emitted by leaves and flowers of *B. nigra* [56,57]. Analogously, a chronic low-level (80 ppb for 5 days) O_3_ exposure did not elicit glucosinolate emissions in different Brassicaceae species [58]. However, it is unclear how glucosinolates might respond to a more severe O_3_ stress and O_3_ exposure combined with drought. The volatile glucosinolate degradation products (isothiocyanates, thiocyanates, nitriles) fulfil an essential role in plant-to-plant and plant-to-insect communication at multiple trophic levels [59,60,61,62,63], and understanding the abiotic stress impacts on these emissions is of major importance for predicting plant communication in multistress natural environments.

Next to *Arabidopsis thaliana*, the classical brassicaceous model, another species, black mustard (*Brassica nigra* (L.) W. D. J. Koch) is giving valuable knowledge to the glucosinolate pathway [64,65,66,67]. *Brassica nigra* is a large plant, 0.5–2 m tall with highly competitive capacity in rural sites; it has a more complex genome and greater tolerance to several environmental stresses than *A. thaliana* [66,67,68]. In a previous study, *B. nigra* had surprisingly high heat stress tolerance and a complex heat stress response [47]. In particular, the heat stress response in *B. nigra* was characterized by different responses of constitutive and stress-induced specialized and generic volatiles and photosynthetic characteristics, including major emissions of glucosinolate breakdown products [47].

The aims of the current study were to investigate how acute and moderate O_3_ exposures alter foliage photosynthetic characteristics and VOC emissions and whether drought modifies O_3_ effects in an interactive manner. We hypothesized that (1) acute O_3_ treatment leads to major reductions in photosynthetic characteristics (*A*, *g*_s_); (2) emissions of specific glucosinolate breakdown products and LOX compounds are enhanced in the recovery phase; (3) the rates of these volatile emissions depend on stomatal O_3_ uptake; (4) drought and O_3_ affect plant volatile emissions in an interactive manner.

## 2. Results

### 2.1. Impacts of Water Stress on Photosynthetic Characteristics and O_3_ Uptake by Leaf Surface and Stomata

In non-ozone-treated plants, net assimilation rate (*A,* mean ± SE) was 15.5 ± 1.7 µmol m^−2^ s^−1^ for well-watered (WW) and 10.9 ± 1.7 µmol m^−2^ s^−1^ for water-stressed (WS) plants and stomatal conductance (*g*_s_) was 300 ± 60 mmol m^−2^ s^−1^ for WW and 107 ± 12 mmol m^−2^ s^−1^ for WS plants. The ratio of intercellular to ambient CO_2_ concentrations (*C*_i_/*C*_a_) in non-ozone-treated WW plants was 0.6–0.8 (*C*_i_ = 261 ± 10 µmol mol^−1^) and 0.2–0.4 (*C*_i_ = 122 ± 8 µmol mol^−1^) for WS plants (all means are different at *p* < 0.001).

**Figure 1:**
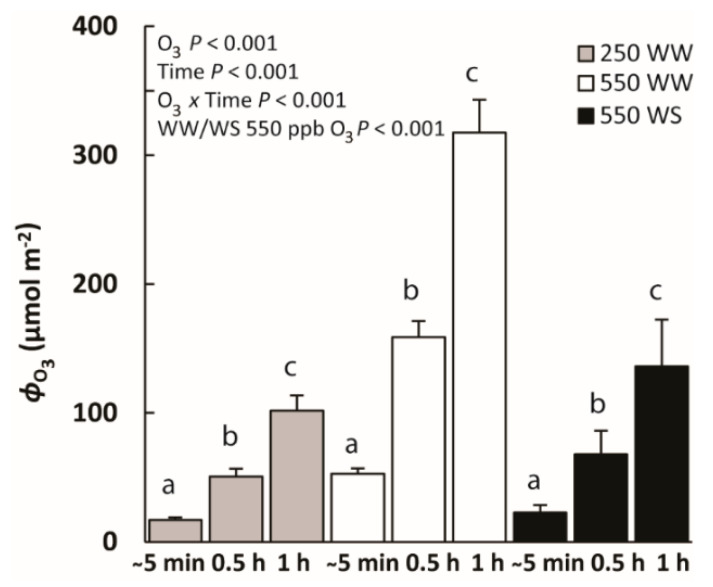
Total O_3_ uptake (*Φ*_O_3__, mean ± SE) in well-watered (WW) plants and water-stressed (WS) *Brassica nigra* plants during the O_3_ treatments of 250 or 550 ppb measured at the beginning (~5 min), in the middle (0.5 h) and at the end (1 h) of the fumigation. Grey bars correspond to WW plants treated with 250 ppb, white bars correspond to WW plants treated with 550 ppb and black bars correspond to WS plants treated with 550 ppb. Data between different time-points within treatments were compared by paired sample *t*-tests and significant differences (*p* < 0.05) are shown by lowercase letters. The effects of exposed O_3_, recovery time and their interaction and the effect of water stress (WS vs. WW) in plants treated with 550 ppb were evaluated by repeated measures ANOVA.

The highest O_3_ uptake was observed in WW plants exposed to 550 ppb O_3_ at all time-points, at the beginning (ca. 5 min from the start of fumigation), in the middle (0.5 h after start of fumigation) and at the end (1 h after the fumigation), reaching 317 ± 25 µmol m^−2^ by the end of the treatment (Figure 1). O_3_ uptake of WW plants exposed to 250 ppb O_3_ and WS plants exposed to 550 ppb O_3_ was similar (Figure 1). O_3_ uptake in these plants varied by only around 16 to 23 µmol m^−2^ at the beginning of the fumigation and around 100 to 140 µmol m^−2^ at the end of the fumigation (Figure 1; O_3_ uptake was significantly greater for WW plants exposed to 550 ppb O_3_ than in the plants in the other two treatments).

At different times during the exposure period, average (±SE) surface O_3_ uptake rate (including uptake by surface and ozone quenching by plant-emitted reactive hydrocarbons) of WW plants exposed to 550 ppb O_3_ (41 ± 5 nmol m^−2^ s^−1^) for the whole exposure period was higher than that of WW plants exposed to 250 ppb O_3_ (13 ± 1 nmol m^−2^ s^−1^; Table 1). For WS plants exposed to 550 ppb O_3_, the surface O_3_ uptake rate was intermediate (25.3 ± 0.4 nmol m^−2^ s^−1^, Table 1).

**Figure 2:**
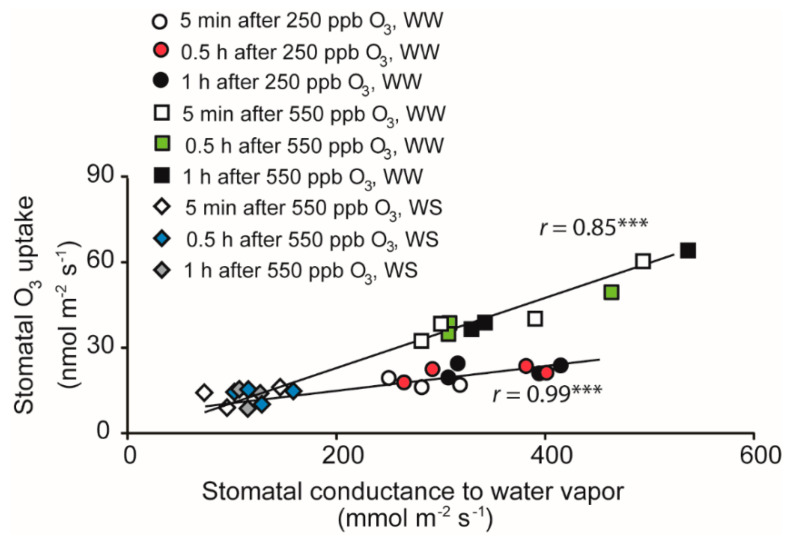
Relationships between stomatal conductance to water vapor and stomatal O_3_ uptake rate in well-watered (WW) *B. nigra* plants treated with 250 or 550 ppb O_3_ and water-stressed (WS) plants treated with 550 ppb O_3_ measured at the beginning (~5 min), in the middle (0.5 h) and at the end (1 h) of the O_3_ exposure. Linear regressions were statistically significant at *p <* 0.001 (***).

Stomatal O_3_ uptake rate in WW plants exposed to 550 ppb O_3_ was higher than in the other two treatments and reached up to 53 ± 4 nmol m^−2^ s^−1^ (Figure 2, Table 1). Stomatal O_3_ uptake rates of WW and WS plants exposed to 250 and 550 ppb O_3_ ranged from 12 to 17 nmol m^−2^ s^−1^ through the exposure period (Table 1). Within treatments, there were only minor time-dependent variations in surface and stomatal O_3_ uptake rate and the ratios of surface and stomatal O_3_ uptake rates to whole leaf uptake rate (Table 1). O_3_ exposure concentration increased surface and stomatal O_3_ uptake rates through all treatments, but the share of surface vs. stomatal uptake rate did not differ significantly among O_3_ concentrations (Table 1). Strong positive relationships between the stomatal O_3_ uptake and *g*_s_ were observed for both 250 ppb and 550 ppb O_3_ fumigations (*p* < 0.001; Figure 2).

### 2.2. Changes in Photosynthetic Characteristics Upon Ozone Exposure

**Figure 3:**
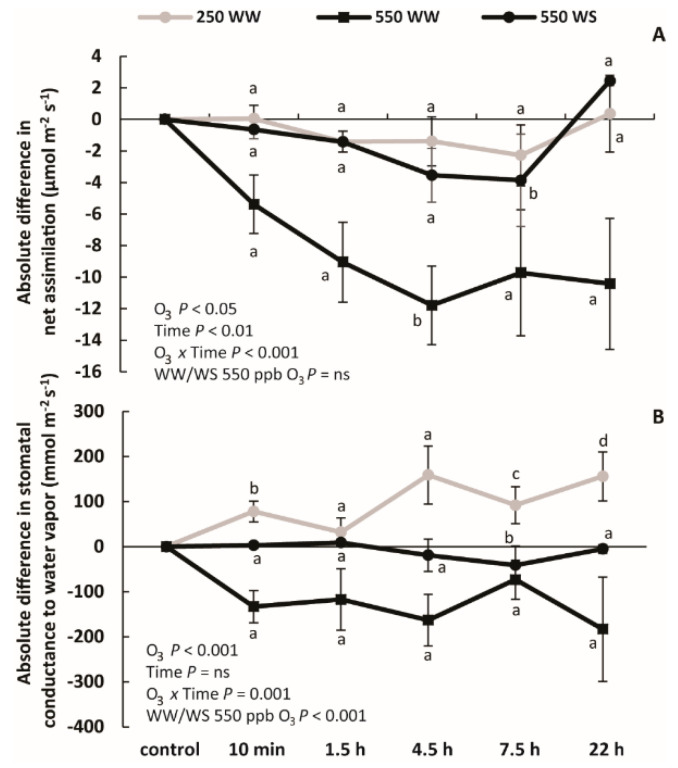
Absolute O_3_-dependent differences (mean ± SE) in net assimilation rate (**A**) and stomatal conductance to water vapor (**B**) in O_3_-treated well-watered and water-stressed *B. nigra* plants. The differences are given as the average values of *A* and *g*_s_ in control plants minus the average values at different time-points during recovery. The values between control and different time-points were compared by paired sample *t*-tests and significant differences (*p* < 0.05) are indicated by lowercase letters. The effects of O_3_ exposure, recovery time, their interaction and the effect of water stress (WS vs. WW) for plants treated with 550 ppb were evaluated by repeated measures ANOVA. ns denotes non-significant effects (*p* > 0.05).

O_3_ exposure reduced *A* in all cases (significant O_3_ effect in Figure 3A) with the reduction observed already 10 min after the exposure to O_3_, followed by a more gradual reduction between 10 min and 4.5 h, and a minor recovery between 7.5 and 22 h for WS plants exposed to 550 ppb O_3_ and WW plants exposed to 250 ppb O_3_, whereas *A* continuously decreased in WW exposed to 550 ppb O_3_ (significant time and O_3_ *x* time effects in Figure 3A). The reduction was greater in plants that took up more O_3_ (Table 1). In particular, at the time point of maximum photosynthetic reduction at 4.5 h, the reduction in *A* was greater in WW plants exposed to 550 ppb O_3_ than that in WS plants exposed to 550 ppb and greater than in WW plants exposed to 250 ppb O_3_ (Figure 3A).

**Figure 4:**
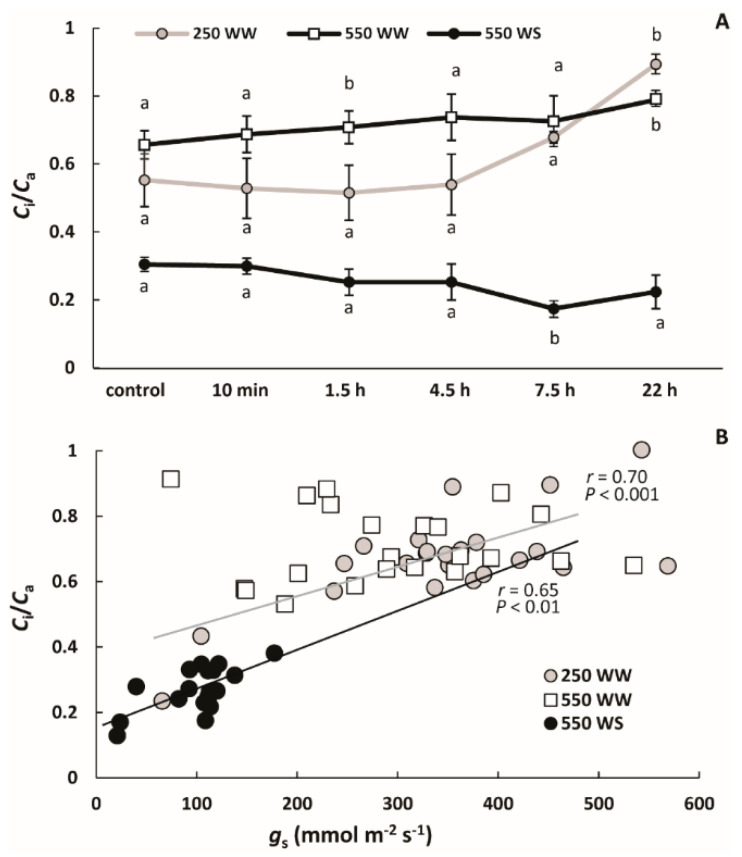
The ratio of mean ± SE intercellular CO_2_ (*C*_i_) to ambient CO_2_ concentration (*C*_i_/*C*_a_; *C*_a_ = 380–400 µmol mol^−1^ during the measurements) after O_3_ fumigation (**A**) and correlations among *C*_i_/*C*_a_ with stomatal conductance to water vapor (*g*_s_) through recovery (10 min, 1.5 h, 4.5 h, 7.5 h and 22 h after O_3_ exposure; (**B**) in *B. nigra*. In A, the data are labelled as: grey continuous line with grey circles—well-watered (WW) plants treated with 250 ppb O_3_; black continuous line with white squares—WW and 550 ppb O_3_; black continuous line with black circles—water-stressed (WS) and 550 ppb O_3_. In B, each data point indicates an individual measurement and the symbols are as: grey circles—WW/250 ppb O_3_; white squares—WW/550 ppb O_3_; black circles—WS/550 ppb O_3_. In **A**, the means at different recovery time-points within treatments were compared by paired sample *t*-tests, significant differences (*p* < 0.05) are indicated by lowercase letters. In **B**, the data were fitted by linear regressions, and the regression lines are shown for the significant relationships (*p* < 0.01 for WW/250 ppb O_3_ (grey line), and WS/550 ppb O_3_ (black line).

Differently from *A*, *g*_s_ showed a decreasing trend only in WW plants exposed to 550 ppb O_3_, although there was a globally significant O_3_ effect across all treatments (Figure 3B). In WS plants exposed to 550 ppb O_3_, *g*_s_ changed little with a significant, but minor reduction, only observed at 7.5 h after O_3_ treatment (Figure 3B). Taken together, 550 ppb O_3_ exposure effect on *g*_s_ was greater for WW than for WS plants (Figure 3B). In the case of WW plants exposed to 250 ppb, *g*_s_ increased during recovery, and *g*_s_ was greater than that in control leaves at the end of the measurements (Figure 3B). Both WW plants exposed to 250 and 550 ppb O_3_ had a higher *C*_i_/*C*_a_ at 22 h recovery point than the controls (Figure 4A). *C*_i_/*C*_a_ in WS plants exposed to 550 ppb was almost constant with a minor reduction at 7.5 h after treatment (Figure 4A). During the recovery period, *C*_i_/*C*_a_ ratio was positively correlated with *g*_s_ in WW plants exposed to 250 ppb O_3_ and WS plants exposed to 550 ppb O_3_ (Figure 4B). *A* values during recovery also correlated positively with *C*_i_/*C*_a_ ratio in WW and WS plants exposed to 550 ppb O_3_ (*p* = 0.02 for both; *p* = 0.09 for WW plants exposed to 250 ppb O_3_; data now shown).

### 2.3. Drought and O_3_ Impacts on Total Volatile Emissions and Emissions of Different Volatile Groups

**Figure 5:**
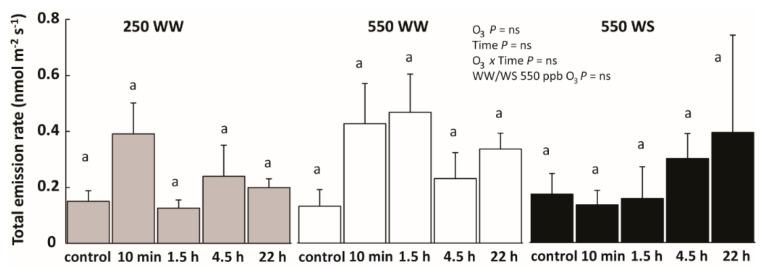
Total volatile emission rates (mean ± SE) in well-watered (WW) and water-stressed (WS) O_3_-fumigated *B. nigra* plants before exposure (control) and through the recovery phase (grey bars—WW/250 ppb O_3_; white bars—WW/550 ppb O_3_; black bars—WS/550 ppb O_3_). The values between different time-points among treatments were compared by paired sample *t*-tests and significant differences (*p* < 0.05) are indicated by lowercase letters. The effect of O_3_ exposure, recovery time, their interaction and the effect of water stress (WS vs. WW) for plants treated with 550 ppb O_3_ were evaluated by repeated measures ANOVA. ns denotes non-significant effects (*p* > 0.05).

**Figure 6:**
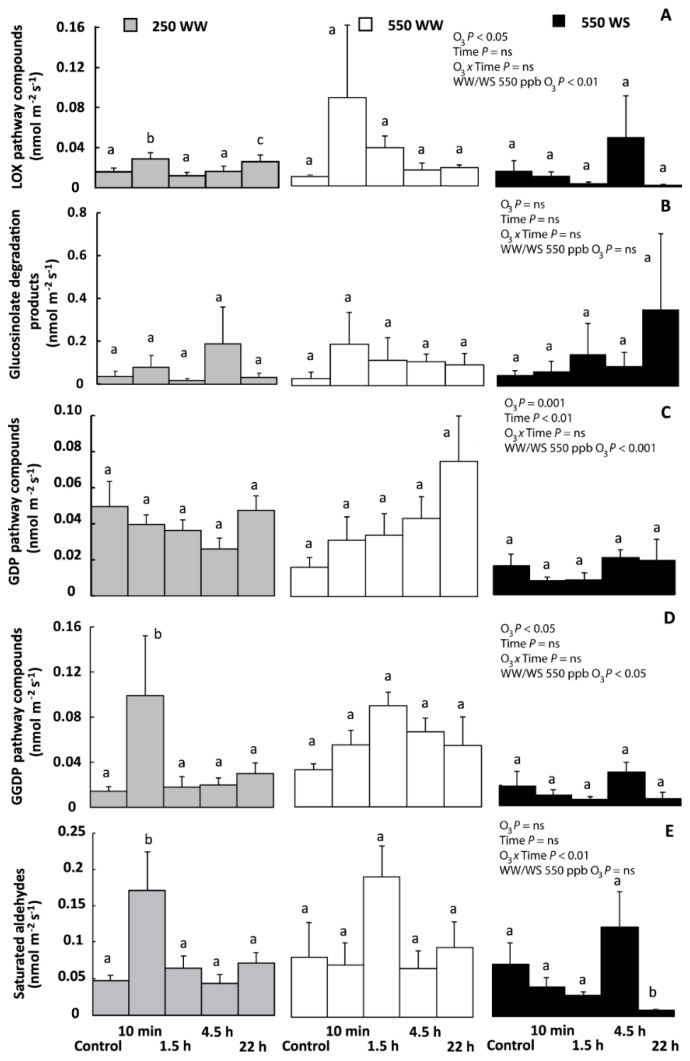
Emission rates (mean ± SE) of volatile lipoxygenase (LOX) pathway compounds (**A**) glucosinolate degradation products; (**B**), geranyl diphosphate (GDP) pathway compounds (monoterpenes; (**C**), geranylgeranyl diphosphate (GGDP) pathway compounds; (**D**), long-chained saturated aldehydes; (**E**) in O_3_-treated well-watered (WW) and water-stressed (WS) *B. nigra* plants before fumigation (control) and through recovery (grey bars—WW/250 ppb O_3_; white bars—WW/550 ppb O_3_; black bars—WS/550 ppb O_3_). Emission rates within treatments at different time-points during recovery were compared by paired sample *t*-tests, and significant differences are indicated by lowercase letters (*p* < 0.05). The effect of O_3_ exposure, recovery time and their interaction and the effect of water stress (WS vs. WW) for plants exposed to 550 ppb O_3_ were evaluated by repeated measures ANOVA. ns denotes non-significant effects (*p* > 0.05). The list of compounds belonging to different volatile groups is shown in Table 2.

In control plants, the total VOC emissions were dominated by saturated aldehydes and geranyl diphosphate (GDP, monoterpenes) or geranylgeranyl diphosphate (GGDP) pathway compounds (carotenoid breakdown products); most WW and WS plants before O_3_ treatment showed similar total VOC emission rates (Figure 5). O_3_ exposure and water stress (for plants exposed to 550 ppb O_3_) modified the share of different volatile groups depending on treatment (Figure 6, see below), but total emissions were not affected (Figure 5).

O_3_ exposure affected all groups of volatiles except glucosinolate breakdown products and saturated aldehydes, but the effects were treatment-specific (Figure 6). LOX compound emissions in WW plants exposed to 250 ppb O_3_ at 10 min and 22 h measurements were enhanced compared to control leaves (Figure 6A). In 550 ppb exposed plants, the LOX compound emissions were greater in WW than in WS plants through the recovery (Figure 6A). For GDP and GGDP compound emissions, O_3_ enhanced emissions, but the emissions at different recovery time points differed little (Figure 6C,D). Only in WW plants exposed to 250 ppb O_3_, the emission of GGDP compounds was greater at 10 min recovery time point compared with the control and other recovery times (Figure 6D). Water stress inhibited GDP and GGDP compound emissions in plants exposed to 550 ppb O_3_ (Figure 6C,D). For saturated aldehydes, the treatments generally had a minor effect. Saturated aldehydes emission was only enhanced in WW plants exposed to 250 ppb O_3_ right after O_3_ exposure, and it decreased in WS plants exposed to 550 ppb O_3_ 22 h after treatment (Figure 6E).

### 2.4. Elicitation of Emissions of Individual LOX Pathway Compounds by O_3_ Exposure

Among the LOX compounds released, pentanal and hexanal were the main compounds in all O_3_ treatments (Table 2). Hexanal emission in WW plants exposed to 550 ppb O_3_ was enhanced at 10 min and 1.5 h recovery times compared to values in control plants (Table 2). In WW plants exposed to 250 ppb O_3_, no other LOX compounds were detected through recovery time, except 1-hexanol emission in one *B. nigra* plant. In WW and WS plants exposed to 550 ppb O_3_, (*Z*)-3-hexen-1-ol and 1-hexanol were the main LOX pathway compounds indicating stress together with 2-ethylfuran, while (*E*)-3-hexen-1-ol, 1-penten-3-ol, (*Z*)-2-penten-1-ol, 1-penten-3-one, (*E,E*)-2,4-hexadienal and 2-methyl-2-cyclopenten-1-one were emitted only at some time-points during recovery (Table 2).

### 2.5. Impact of Glucosinolate Breakdown Products on the O_3_-Induced Smell Bouquet

All control and stressed plants released a similar blend of glucosinolate breakdown products 22 h after the O_3_ treatments (Monte Carlo permutation test, *p* > 0.05, data not shown) (Table 2). Specific compounds like dimethyl disulfide and methanethiol were characteristic to WW plants exposed to 250 ppb O_3_ and WS plants exposed to 550 ppb O_3_, but they were rare in WW plants exposed to 550 ppb O_3_ (Table 2). The rest of the volatile glucosinolates, cyclohexyl isocyanate, cyclohexyl isothiocyanate, 2-propenenitrile, tetramethylthiourea and tetramethylurea, were characteristic to the control—as well as the stressed plants of different treatments (Table 2).

### 2.6. O_3_ Exposure Effects on Other Volatiles

Among GDP pathway compounds, at 4.5 h recovery point, α-pinene emission was decreased in WW plants exposed to 250 ppb O_3_ and at 22 h recovery point, 3-carene emission was enhanced in WW plants exposed to 550 ppb O_3_ compared to the values in control plants (Table 2). Among saturated aldehydes, decanal, heptanal, nonanal and octanal emissions were enhanced in WW plants exposed to 250 ppb O_3_ right after exposure compared with the emissions in controls (Table 2). Heptanal emission was greater at 4.5 h recovery time in WS plants exposed to 550 ppb O_3_ than in the controls (Table 2).

## 3. Discussion

### 3.1. How Drought and Different O_3_ Levels Affect Leaf Photosynthetic Characteristics in B. nigra

The entry of gaseous pollutants into the leaf interior is controlled by the openness of stomatal pores and stomatal density that together determine the stomatal conductance and the diffusion of the given pollutant into leaf interior (Figure 1) [69,70,71]. Before O_3_ treatment, drought stress strongly reduced the stomatal conductance (*g*_s_, Figure 2 and Figure 4), and the ratio of intercellular CO_2_ concentration (*C*_i_) to ambient CO_2_ concentration (*C*_a_) (*C*_i_/*C*_a_) (Figure 4) in water-stressed (WS) plants compared with well-watered (WW) plants, highlighting the classical plant response to drought that allows conservation of water use [41,72,73,74]. Apart from drought effects, O_3_ typically triggers a rapid stomatal closure and the production of reactive oxygen species (ROS) [16,70,75,76,77]. In our study, in WW plants exposed to 550 ppb O_3_, stomatal closure was already observed at 10 min after O_3_ treatment, and the stomata remained closed through the recovery period (Figure 3B). Despite stomatal closure, due to higher initial *g*_s_ and high O_3_ exposure dose, the highest stomatal O_3_ uptake rate was observed in this treatment (Figure 1, Figure 2 and Figure 3, Table 1). In contrast, there was almost no effect of O_3_ on *g*_s_ in WS plants exposed to 550 ppb O_3_, except at 7.5 h of the recovery period and there was a moderate increase in WW plants exposed to 250 ppb O_3_ at the end of the recovery period (Figure 3B). Small to moderate impacts of O_3_ treatments on *g*_s_ in WS plants exposed to 550 ppb and WW plants exposed to 250 ppb indicate that O_3_ constituted a mild stress in these treatments. These data collectively suggest that *B. nigra* is a relatively ozone-tolerant species as generally observed for cruciferous plants [68,78,79].

Abscisic acid (ABA) is considered to be the most important chemical regulator of stomatal functioning in plants [30], and these differences among treatments might be related to differences in ABA accumulation and stomatal sensitivity to ABA. Under drought stress, the stomatal closure is strongly dependent on ABA, and the ABA-sensitivity increases with the severity of drought stress [30,80]. O_3_, in turn, reduces the stomatal sensitivity to ABA [81], and this can ultimately lead to a failure to close stomata, also called stomatal sluggishness [40], in response to environmental stimuli such as reduced humidity or reduced light characteristically decreasing stomatal conductance [82]. Thus, the reduction of ABA sensitivity might explain why in WS plants *g*_s_ was reduced only to a minor degree upon O_3_ exposure, and the stomatal response was delayed until 7.5 h after O_3_ exposure, indicative of stomatal sluggishness (Figure 3B and Figure 4B). Despite the reduction in stomatal sensitivity, water stress-driven stomatal closure strongly reduced the amount of O_3_ entering through the stomata (Table 1, Figure 2). Similar results have been reported in water-stressed and O_3_-treated *Phaseolus vulgaris* [44] and *Quercus ilex* [83]. As the result of limited O_3_ entry, water stress diminishes O_3_ damage in plants [36,84,85]. Yet, in the long term, water stress inevitably reduces photosynthetic production and plant biomass, and this can be exacerbated by ozone stress [83].

Our study demonstrated a classic reduction of photosynthetic rate (*A*) by water stress due to diminished *g*_s_ and CO_2_ availability (Figure 3 and Figure 4) as observed in numerous studies [26,36,86], and this reduction was only moderately enhanced by O_3_ exposure (Figure 3A). This further emphasizes that reduced *g*_s_ prior to O_3_ exposure protects photosynthetic machinery and results in a lower effective O_3_ dose as observed also in other studies [23]. Similarly to WS plants exposed to 550 ppb O_3_, exposure of WW plants to 250 ppb O_3_ caused only a minor reduction in *A* (Figure 3A). Although the reductions in *A* and *g*_s_ were apparently correlated (Figure 3) as demonstrated by strong positive relationships *C*_i_/*C*_a_ ratio and *g*_s_ (Figure 4B), *g*_s_ and *A* changed differently during recovery after O_3_ exposure. Indeed, the *C*_i_/*C*_a_ ratio increased by the end of the recovery period in WW plants (Figure 4A), but due to different reasons for plants exposed to 250 ppb and 550 ppb O_3_. In the case of 250 ppb O_3_ fumigation, *A* remained at the control level and *g*_s_ was increased, whereas in the case of 550 ppb O_3_ fumigation, both *A* and *g*_s_ were reduced, but the reduction in *A* was greater (Figure 3). This indicates that non-stomatal factors also affected *A* in WW plants exposed to the higher O_3_ concentration. Such non-stomatal factors responsible for the decrease of *A* can be the reduction in ribulose 1,5-bisphosphate (RuBP) carboxylase/oxygenase activity, inactivation of photosynthetic electron transport and concomitant reduction in RuBP regeneration rate [87,88]. Analogously to our study, a reduction of *A* partly independent of *g*_s_ has been shown in other species [89,90]. Furthermore, as a reduction in *A* can also cause a reduction in *g*_s_ [91,92], we cannot rule out the non-stomatal reduction in *A* in earlier phases of recovery in WW plants exposed to 550 ppb O_3_.

### 3.2. Water Stress Effects on Non-Ozonated Plant Volatile Emissions

Plant physiological and metabolic processes critically depend on water stress duration and severity [40,93,94,95]. Mild water stress can have only limited effects on constitutive VOC emissions, but moderate water stress might enhance VOC emissions, and severe water stress can decrease the emissions [35,40,96,97]. Although water stress might alter the constitutive VOC emissions, it typically does not result in induction of stress volatile emissions [98]. In our study, water stress did not affect significantly the total average VOC emission rate and the emission rate and chemical composition of volatiles was similar among non-ozonated WW and WS *B. nigra* plants (Figure 5, Table 2). Given that the constitutive VOC emissions occur at a low level in *B. nigra* (Figure 5, Table 2) [47], the metabolic energy and photosynthetic substrate requirements for VOC synthesis are also low. Therefore, it is plausible that the reduction in photosynthate production rate in water-stressed plants did not limit the substrate availability for constitutive volatile synthesis in *B. nigra*.

### 3.3. O_3_ Effects on Total VOC Emissions in Well-Watered and Water-Stressed Plants

In the present study, both WW and WS plants released stress VOCs after the treatment with 550 ppb O_3_ (Table 2). The blend of released volatiles was similar to that in the heat-stressed *B. nigra* [47], yet the different treatments in our study did not affect the total emissions of VOCs (see Results). Similarly, limited effects of chronic low-level O_3_ treatments on VOC emissions in *B. nigra* have been observed in other studies [57,99]. Nevertheless, the emission of VOCs from plants is affected by the stress dose (severity) and also depends on plant species [40,47,93,94,100,101]. In another O_3_ treatment study, *B. nigra* was most ozone tolerant compared to *Sinapis alba*, *S. arvensis* and *B. napus* [57].

### 3.4. Effects of O_3_ and Water Stress Treatments on Emissions of Specific LOX Pathway Compounds

Presence of LOX compounds in the emission blend typically indicates severe stress; LOX emissions can be induced upon mechanical damage, heat, ozone, and herbivory stresses, and the rate of LOX compound emissions often scales with the severity of stress [20,102,103,104]. O_3_ exposure enhanced LOX compounds emission across all treatments, but comparison of WS and WW 550 ppb O_3_-treated plants, demonstrated that WW plants reacted more strongly (significant water stress effect; Figure 6A). In WS plants, less O_3_ entered the leaves compared to WW plants exposed to 550 ppb O_3_ and, as a result, the O_3_-dependent increase in total LOX emissions was absent, although stress indicator compounds were detected (Table 1 and Table 2, Figure 6A). There is also a probability that we might have lost some LOX compound emissions during the first hour of O_3_ treatment in WW plants because the earliest LOX emission burst can occur a few minutes to 30 min after the damage of plant cells [105,106,107]. On the other hand, LOX-related volatiles emerge 1–2 h [23] or in some cases even 5 h after the O_3_ fumigation [20]. Clearly, we have been able to detect the delayed LOX emission response in WW plants exposed to 250 and 550 ppb O_3_ (Figure 6A).

In our study, hexanal and pentanal were the main LOX volatiles forming the vast amount of the LOX compounds emitted upon O_3_ exposure (Table 2). The same LOX compound composition was observed in O_3_-treated *N. tabacum* [89]. The detection of various LOX pathway compounds in the emissions of WW or WS *B. nigra* plants during recovery confirms the presence of cell membrane damage [108,109]. LOX pathway volatiles like (*Z*)-3-hexen-1-ol, 1-hexanol, 1-penten-3-ol, 1-penten-3-one together with 2-ethylfuran (Table 2) were characteristic only to the WW and WS plants exposed to 550 ppb O_3_. Next to typical LOX compounds, we have observed previously that 2-ethylfuran is one of the signals of a severe stress as its emissions increased in *N. tabacum* and *B. nigra* after a threshold temperature was exceeded [47,104]. It is proposed that 2-ethylfuran is formed from (*E*)-2-hexenal and co-emitted with (*Z*)-3-hexenol [110,111], and 2-ethylfuran was observed in WW 550 ppb O_3_-treated plants in our study as well. This evidence collectively suggests that at a given O_3_ exposure, higher stomatal O_3_ uptake caused a more severe stress of WW *B. nigra* plants (Table 1 and Table 2; Figure 1 and Figure 2).

### 3.5. O_3_ Effects on Species—Specific Glucosinolate Degradation Products

Glucosinolates and/or their degradation products are characteristic compounds of the Brassicaceae that are considered as human health-promoting compounds and having an important role in plant protection [112,113]. The results of this study showed that O_3_ or water stress did not significantly affect emissions of glucosinolate degradation products (Figure 6B). Nevertheless, we detected multiple glucosinolate breakdown products in WW and WS plants during the recovery period, including damage-related dimethyl disulfide and even methanethiol [114,115] (Table 2). Lack of a quantitative scaling of glucosinolate breakdown product emission and O_3_ exposure might indicate that O_3_ treatments did not cause extensive cell damage that would have led to enhanced activation of the glucosinolate–myrosinase system [49,116]. This result is different from heat-stressed *B. nigra*, where emissions of glucosinolate breakdown products and LOX compounds occurred similarly [47]. Over the longer term, O_3_ treatment and water-deficit can lead to modifications in glucosinolate content [34,54,96], and this could further contribute to alterations in the release of glucosinolate breakdown products under O_3_ or other stresses.

### 3.6. O_3_ Effects on Other Volatile Groups

The plastidial methylerythritol phosphate (MEP) pathway is responsible for the biosynthesis of GDP-derived compounds and their emission depends on the supply of photosynthates [117,118]. Being highly reactive, monoterpenes could improve plant thermo- or O_3_-tolerance by reacting with stress-generated oxidative compounds [119,120]. In the present study, the emission of GDP compounds through recovery was similar within O_3_ treatments (Figure 6C). Such a limited time-dependent response might seem surprising given the strong changes in photosynthetic rate (Figure 3). However, this might indicate the overall low GDP compound emission rate in *B. nigra* such that the carbon flux going into GDP compound synthesis is small and not suppressed by the O_3_-driven reductions in photosynthesis. Decreased GDP emissions have been previously found in O_3_ treated *N. tabacum* [89] and *B. napus* [121], and relatively stable GDP emissions in O_3_ treated *B. nigra* plants [56,89,121,122,123].

Part of our results, especially high GGDP compounds emission at 10 min recovery time in WW plants exposed to 250 ppb O_3_, support earlier findings of increased emission of GGDP compounds upon O_3_ treatment [38,124,125]. It has been suggested, that in *B. nigra* the release of GGDP compounds is related to the oxidative cleavage of carotenoids [126,127]. Carotenoids are continuously produced and degraded in green plants [128,129], but their primary role is to protect the photosynthetic apparatus against the oxidative stress [130,131].

The release of saturated aldehydes is probably related to several pathways functioning simultaneously with the LOX pathway such as the conversion of saturated fatty acids into aldehydes or the opposite [109,132,133]. Emission of saturated aldehydes could be partly controlled by stomatal conductance [43,134], but in our study there was no evidence of the direct effect of stomata on the emissions of these volatiles through recovery in different treatments and an overall limited effect of different treatments on saturated aldehyde emissions (Figure 6E). Decanal and nonanal are commonly found in the odor of *B. nigra* leaves and flowers and more importantly, their emission is associated with plant O_3_ tolerance and O_3_ dose, and the degree of damage [135,136,137]. For example, *B. nigra* plants treated with 70 ppb or 120 ppb O_3_, showed diminished emissions of nonanal and decanal, while the treatment of *B. napus* leaves with ~140 ppb O_3_ increased the release of aforementioned saturated aldehydes [56,121]. The insensitivity of saturated aldehyde emissions to water stress and O_3_ exposure further underscores the high O_3_ tolerance of *B. nigra*.

## 4. Materials and Methods

### 4.1. Plant Material

**Figure 7:**
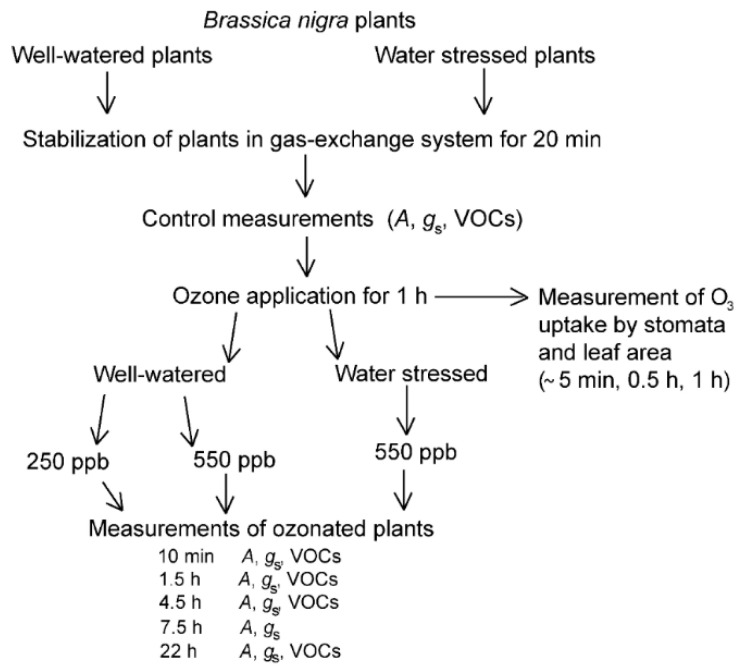
Scheme of studying ozone stress of well-watered and water-stressed plants of *Brassica nigra*. After measurements of photosynthetic characteristics and emissions of volatile organic compounds (VOCs) of control plants, well-watered *B. nigra* plants were treated with O_3_ concentrations of 250 ppb and 550 ppb, while water-stressed plants were only exposed to 550 ppb. The exposure period was 1 h and O_3_ uptake by stomata and leaf surface was recorded during the exposure. Photosynthetic characteristics were measured 10 min, 1.5 h, 4.5 h, 7.5 h and 22 h after O_3_ stress. In the case of VOCs, measurements at 7.5 h were not conducted.

*Brassica nigra* seeds of local source were purchased from the Department of Entomology, University of Wageningen, the Netherlands. Seeds were sown in 0.8 L plastic pots filled with commercial soil (Biolan Oy, Eura, Finland) and quartz sand (1:1 mixture) (AS Silikaat, Tallinn, Estonia). The soil was fertilized with a slow-release mineral fertilizer at an optimum level (Biolan Oy, Eura, Finland). Plants were grown at a light intensity of 400 µmol m^−2^ s^−1^ (HPI-T Plus 400 W metal halide lamps, Philips, Brussels, Belgium), relative humidity of 60%, day length of 12 h and day/night temperatures of 24/20 °C in a plant growth room. During the first three weeks, the plants were watered every other day and at the beginning of the fifth week. Then the plants were randomly divided between ‘well-watered plants’ (WW) and ‘water-stressed plants’ (WS; Figure 7). Watering of WW plants was continued as described above, but WS plants were watered once a week, resulting in a mild water stress as plant water potential was −0.5 to −0.8 MPa (measured with a PMS 600 pressure chamber, PMS Instrument Company, Albany, OR, USA). The experiment was conducted with mature fully-expanded leaves (ca. 3 week old leaves).

### 4.2. Experimental Set-Up and Gas Exchange Measurements

A custom-made gas-exchange system was used for foliage photosynthesis and transpiration measurements and volatile sampling. The system has a 1.2 L temperature-controlled double-walled glass chamber that is connected to an infra-red dual-channel gas analyzer (CIRAS II, PP-Systems, Amesbury, MA, USA) for measuring CO_2_ and H_2_O concentrations at the chamber in- and outlets [138]. Chamber temperature is regulated by a controlled-temperature water bath that circulates water between the double glass walls of the gas-exchange chamber [138]. All gas-exchange measurements were conducted at standard conditions of light intensity of 800 µmol m^−2^ s^−1^ at leaf surface, air temperature inside the chamber of 25 °C (leaf temperature was ± 1 °C of chamber temperature) and relative humidity 60%. The temperature inside the gas-exchange chamber was monitored by a thermistor (NTC thermistor, model ACC-001, RTI Electronics, Inc., St. Anaheim, CA, USA). The air was taken from outside, passed through a 10 L buffer volume, an HCl-activated copper tubing for scrubbing O_3_ and further through a humidifier. Inside the chamber, the CO_2_ concentration was 380–400 µmol mol^−1^ and the air flow rate was set to 1.6 L min^−1^. Turbulent conditions inside the chamber were achieved by a fan installed in the chamber. Three fully-expanded mature upper canopy leaves were inserted in the chamber, and 20–30 min after plant enclosure, when leaf gas-exchange rates had stabilized, net assimilation rate (*A*) and stomatal conductance to water vapor (*g*_s_) were recorded (Figure 7). Simultaneously with the gas-exchange measurements, volatiles were collected as described below. Values of *A* and *g*_s_ were calculated according to [139].

### 4.3. Ozone Stress Application

O_3_ concentration in chamber in- and outlets was monitored by a UV photometric O_3_ detector (Model 49i, Thermo Fisher Scientific, Waltham, MA, USA). In the ambient air entering the chamber, O_3_ concentration was less than 2 ppb. O_3_ was generated with a Stable Ozone Generator SOG-2 (LLC-Upland, CA, USA) and mixed with the air entering the chamber to the desired concentration. Plants were exposed to O_3_ for 1 h as follows: 250 ± 10 ppb for 6 WW plants and 550 ± 25 ppb for 5 WW plants and 4 WS plants (Figure 7). Exposure with 250 ppb O_3_ was not tested for WS treatment as preliminary experiments showed no effect on volatile emissions due to too low O_3_ uptake. *A* and *g*_s_ were recorded 10 min, 1.5 h, 4.5 h, 7.5 h and 22 h after O_3_ exposure, and volatiles were collected 10 min, 1.5 h, 4.5 h and 22 h after O_3_ treatment. O_3_ uptake rate by stomata (nmol m^−2^ s^−1^), leaf surface (nmol m^−2^ s^−1^) and the total amount of O_3_ uptake (*Φ*_O_3__, µmol m^−2^) by the leaves were calculated at the beginning (~5 min), in the middle (0.5 h) and at the end (1 h) of O_3_ application by using the O_3_ binary diffusion coefficient and equations from [23]. “Surface” ozone uptake also includes quenching of ozone by reactive hydrocarbons emitted by leaves [89,140].

### 4.4. Volatile Sampling and GC-MS Analysis

Multi-bed stainless steel cartridges filled with three different carbon-based adsorbents were used for collecting the volatiles (VOCs) [141]. A portable 210-1003MTX air sampling pump (SKC Inc., Houston, TX, USA) was connected to the chamber outlet with a T-piece and the air enriched with plant VOCs was drawn from the chamber through the cartridge with a constant flow rate of 200 mL min^−1^ for 20 min. Each day before the plant measurements, VOCs in the empty chamber were collected and subtracted from measurements with plants.

The cartridges were analyzed with a combined Shimadzu TD20 automated cartridge desorber connected to a Shimadzu 2010 Plus gas chromatograph-mass spectrometer (GC–MS) (Shimadzu Corporation, Kyoto, Japan). Adsorbent cartridges were heated to 250 °C and back-flushed with high purity He (99.9999% AGA, The Linde Group, Tallinn, Estonia) at a flow rate of 40 mL min^−1^ for 6 min. During this period, desorbed VOCs were collected onto a cold trap filled with Tenax TA at −20 °C. In the second stage, the trap was heated to 280 °C and during 6 min, VOCs were carried with He into a Zebron ZB-624 fused silica capillary column (0.32 mm i.d., 60 m, 1.8 μm film thickness, Phenomenex, Torrance, CA, USA). In GC, the flow rate of the carrier gas (He) was 2.9 mL min^−1^ and VOCs were separated according to the following program: oven temperature kept at 40 °C for 1 min, then increasing the temperature by 9 °C min^−1^ to 120 °C, held for 5 min, then increasing the temperature by 2 °C min^−1^ to 190 °C, held for 2 min, and finally increasing the temperature by 5 °C min^−1^ to 250 °C, held for 5 min. The Shimadzu QP2010 Plus mass spectrometer (MS) was operated in the electron impact mode. The transfer line temperature was 255 °C and ion-source temperature 170 °C. The GC-MS system was calibrated as explained in [47] and in [141]. VOCs (Table 1) were identified by comparing their mass spectra to the spectra of authentic standards and to those in the NIST library (NIST05). Volatile emissions rates per leaf area were calculated according to [142].

Volatiles were divided among five groups according to their biosynthesis pathways (Table 2). Group 1: lipoxygenase pathway (LOX) volatiles such as (*Z*)-3-hexen-1-ol, 1-hexanol, hexanal, 1-penten-3-ol, pentanal etc. together with 2-ethylfuran and 2-methyl-2-cyclopenten-1-one; Group 2: glucosinolate degradation products such as 2-propenenitrile, cyclohexyl isocyanate, cyclohexyl isothiocyanate, dimethyl disulfide, methanethiol, methyl isothiocyanate, tetramethylthiourea and tetramethylurea; Group 3: geranyl diphosphate (GDP) pathway volatiles (monoterpenes) α- and β-pinene, 3-carene, limonene, and camphene; Group 4: geranylgeranyl pathway (GGDP) volatiles (carotenoid degradation products) 6-methyl-5-hepten-2-one and geranyl acetone; Group 5: long-chained saturated aldehydes (≥C7) heptanal, octanal, nonanal and decanal.

### 4.5. Statistical Analyses

For statistical analyses, net assimilation rate (*A*) and stomatal conductance (*g*_s_) were log-transformed. The controls for WW and WS plants (values prior to O_3_ exposure) were compared with the values of treated plants at different time-points by paired sample *t*-tests. The differences of *A* and *g*_s_ between the control and treated plants were calculated as *V*_c_-*V*_t_(*t*), where *V*_c_ is the average trait value of control and *V*_t_ is the average trait value of O_3_-treated plants at time *t* after O_3_ treatment. Paired sample *t*-tests were used to compare the paired values of photosynthetic characteristics, ozone uptake characteristics and log-transformed total VOC emission rates and average emission rates at different time-points during recovery within the given treatment.

The effects of O_3_, recovery time, their interaction and the effect of water stress (for WW and WS plants exposed to 550 ppb O_3_) on *A*, *g*_s_, total VOC emission, VOC groups and O_3_ uptake rates by stomata, leaf surface and total O_3_ uptake were evaluated by repeated measures ANOVA. Linear regression analyses were used to explore the relationships of stomatal O_3_ uptake with *g*_s_, and the relationship of the *C*_i_/*C*_a_ ratio with *g*_s_ during recovery. All these statistical analyses were conducted with STATISTICA 7 (StatSoft Inc., Tulsa, OK, USA). All statistical effects were considered significant at *p* < 0.05.

Differences in volatile emissions for different combinations of O_3_ exposure and water stress were evaluated by principal component analyses (PCA) [143] using CANOCO 5.0 software (ter Braak and Smilauer, Biometris—Plant Research International, the Netherlands). In total, 71% of data variance was explained by the first and the second principal components (PCA 1 46%, PCA 2 25%) (data not shown). Before the analyses, the data were mean-centered and log-transformed. Redundancy data analysis (RDA) with the Monte-Carlo permutation test was used to test for statistical differences in emission blends across the treatments and no statistical differences were observed (*p* > 0.05, data not shown).

## 5. Conclusions and Outlook

This study demonstrated classic reductions in stomatal conductance (*g*_s_) and net assimilation rate (*A*) in water-stressed *B. nigra*, but the impact of water stress on constitutive volatile emissions was minor, nor did water stress elicit emissions of stress volatiles. In well-watered plants, acute O_3_ stress (550 ppb exposure for 1 h) led to major reductions in *g*_s_ and *A* with limited recovery by the end of the experiment at 22 h. In these plants, acute O_3_ stress also led to induction of emissions of lipoxygenase pathway (LOX) volatiles between 10 min and 1.5 h after exposure. Due to lower *g*_s_, O_3_ uptake was reduced under water stress, and as a result, soil water limitation strongly ameliorated the impact of acute O_3_ stress, as evidenced in much lower reductions in *g*_s_ and *A* and lack of induction of LOX volatiles. Ozone uptake was similar in well-watered plants exposed to a lower O_3_ concentration of 250 ppb and in water-stressed plants, and the responses of foliage physiological characteristics to O_3_ exposure were also similar, except that *g*_s_ and *A* were maintained at a higher level in well-watered plants. This suggests that water stress did not result in the priming of plants to subsequent O_3_ stress. Nevertheless, O_3_ exposure resulted in surprisingly minor modifications in volatile profiles in the annual plant *B. nigra* compared with observations for perennial species. This might be indicative of high ozone tolerance of *B. nigra*, but also reflect the general strategy of annual species that have a rapid leaf turnover and, instead of responding stronger after the stress and repairing the damages, might sacrifice the heavily damaged leaves to form new leaves [144]. In the current study, we looked at O_3_ responses right after exposure and through 22 h recovery, but O_3_ exposure can have longer-term effects, including initiation of acclimation responses that improve plant tolerance to sustained mild chronic O_3_ stress [145,146]. We suggest that future studies should look at stress memory and priming effects including alterations in the physiological characteristics of O_3_-stressed leaves and new leaves formed after O_3_ stress.

## Figures and Tables

**Table 1:** Average surface and stomatal O_3_ uptake rates and relative uptake rates at the beginning (~5 min), in the middle (0.5 h) and at the end (1 h) of fumigation with 250 ppb or 550 ppb O_3_ for well-watered (WW) and water-stressed (WS) *Brassica nigra* plants.

Uptake Rate	WW/250 ppb O_3_			WW/550 ppb O_3_			WS/550 ppb O_3_			*p* Value		
	Time			Time			Time					
	~5 min	0.5 h	1 h	~5 min	0.5 h	1 h	~5 min	0.5 h	1 h	O_3_	Time	O_3_ *x* Time
Surface O_3_ uptake rate (nmol m^−2^ s^−1^**)**	15.6 ± 2.4 ^a^	12.1 ± 2.0 ^b^	11.5 ± 2.2 ^b^	43.1 ± 4.0 ^a^	35.8 ± 4.1 ^a^	44 ± 8 ^a^	26 ± 9 ^a^	25 ± 9 ^a^	25 ± 9 ^a^	<0.01	<0.05	ns
Stomatal O_3_ uptake rate (nmol m^−2^ s^−1^)	13.1 ± 2.8 ^a^	16.1 ± 3.1 ^b^	16.8 ± 3.5 ^b^	54 ± 11 ^a^	60 ± 12 ^a^	44 ± 5 ^a^	12 ± 2 ^a^	13.3 ± 1.7 ^a^	12.4 ± 1.4 ^a^	0.001	ns	<0.05
Surface O_3_ uptake rate/Whole leaf uptake rate (%)	56 ± 8 ^a^	45 ± 8 ^b^	43 ± 10 ^b^	50.5 ± 2.8 ^a^	43 ± 6 ^a^	50 ± 7 ^a^	66 ± 5 ^a^	61 ± 7 ^a^	63 ± 8 ^a^	ns	0.01	ns
Stomatal O_3_ uptake rate/Whole leaf uptake rate (%)	44 ± 8 ^a^	55 ± 8 ^b^	57 ± 10 ^b^	49.5 ± 2.8 ^a^	57 ± 6 ^a^	50 ± 7 ^a^	34 ± 5 ^a^	39 ± 7 ^a^	37 ± 8 ^a^	ns	<0.05	<0.05

Differences between ~5 min and other time-points were separated by paired samples *t*-tests, and differences significant at *p* < 0.05 are shown by lowercase letters. The effect of O_3_ exposure, recovery time and their interaction were evaluated by repeated measures ANOVA. ns denotes non-significant effects (*p* > 0.05).

**Table 2:** Average (±SE) emission rates of individual volatile organic compounds detected in the emissions of well-watered (WW) and water-stressed control and O_3_-treated (250 ppb or 550 ppb O_3_ for 1 h) *Brassica nigra* plants.

		WW/250 ppb O_3_					WW/550 ppb O_3_					WS/550 ppb O_3_				
	Compound	Control	10 min	1.5 h	4.5 h	22 h	Control	10 min	1.5 h	4.5 h	22 h	Control	10 min	1.5 h	4.5 h	22 h
LOX pathway																
**1**	2-Ethylfuran							0.015 ± 0.013		0.018 *			0.008 ± 0.007 ^a^		0.02 ± 0.01 ^a^	
**2**	(*E, E*)-2,4-Hexadienal							0.024								
**3**	Hexanal	0.0090 ± 0.0028 ^a^	0.018 ± 0.005 ^a^	0.0070 ± 0.0018 ^a^	0.0098 ± 0.0030 ^a^	0.015 ± 0.005 ^a^	0.0030 ± 0.0012 ^a^	0.010 ± 0.004 ^b^	0.018 ± 0.007 ^c^	0.0064 ± 0.0020 ^a^	0.0128 ± 0.0007 ^a^	0.011 ± 0.008 ^a^	0.0028 ± 0.0005 ^a^	0.0017 ± 0.0004 ^a^	0.0086 ± 0.0034 ^a^	0.00070 ± 0.00050 ^a^
**4**	1-Hexanol		0.0014					0.018	0.0022 ± 0.0003				0.0015		0.0036 ± 0.0030	
**5**	(*E*)-3-hexen-1-ol							0.180								
**6**	(*Z*)-3-hexen-1-ol							0.010	0.03 ± 0.01	0.002			0.0197		0.027 ± 0.025	
**7**	2-Methyl-2-cyclopenten-1-one							0.012								
**8**	Pentanal	0.0068 ± 0.0014 ^a^	0.010 ± 0.002 ^a^	0.0039 ± 0.0013 ^a^	0.0070 ± 0.0024 ^a^	0.0103 ± 0.0017 ^a^	0.0062 ± 0.0015 ^a^	0.0063 ± 0.0013 ^a^	0.0042± 0.002 ^a^	0.0026 ± 0.0010 ^a^	0.0070 ± 0.0026 ^a^	0.0048 ± 0.0026 ^a^	0.0023 ± 0.0005 ^a^	0.0026 ± 0.0007 ^a^	0.0018 ± 0.0010 ^a^	0.00119 ± 0.00003 ^a^
**9**	1-Penten-3-ol							0.034							0.022	
**10**	(*Z*)-2-Penten-1-ol							0.053								
**11**	1-Penten-3-one									0.016						
Glucosinolate degradation products																
**12**	Cyclohexyl isocyanate	0.0087 ± 0.0029 ^a^	0.053	0.0008	0.070 ± 0.066 ^a^	0.0176 ± 0.0032 ^a^	0.0073 ± 0.0040 ^a^	0.030 ± 0.020 ^a^	0.034 ± 0.030 ^a^	0.0083 ± 0.0050 ^a^	0.0097 ± 0.0010 ^a^	0.012 ± 0.007 ^a^	0.0015 ± 0.0004 ^a^	0.0011 ± 0.0003 ^a^	0.0050 ± 0.0027 ^a^	0.16
**13**	Cyclohexyl isothiocyanate	0.0017 ± 0.0011 ^a^	0.005 ± 0.004 ^a^	0.00005	0.006 ± 0.005 ^a^	0.00121± 0.00040 ^a^	0.003	0.0062 ± 0.0040 ^a^	0.0031± 0.0029 ^a^	0.009	0.00305 ± 0.00006 ^a^	0.0017 ± 0.0006 ^a^	0.00111 ± 0.00020 ^a^	0.0019 ± 0.0005 ^a^	0.0015 ± 0.0005 ^a^	0.09
**14**	Dimethyl disulfide		0.004	0.0031 ± 0.0010 ^a^	0.0017 ± 0.0012 ^a^	0.0007				0.003	0.011		0.0004	0.00023 ± 0.00007 ^a^		
**15**	Methanethiol	0.0056 ± 0.0034 ^a^	0.003	0.016 ± 0.013 ^a^	0.0080						0.013	0.0011	0.0003			0.003
**16**	2-Propenenitrile	0.09 ± 0.08 ^a^	0.036 ± 0.018 ^a^		0.18 ± 0.16 ^a^	0.043 ± 0.020 ^a^	0.04	0.24 ± 0.17 ^a^	0.18 ± 0.15 ^a^	0.084 ± 0.040 ^a^	0.09 ± 0.05 ^a^	0.053 ± 0.020 ^a^	0.08 ± 0.06 ^a^	0.22 ± 0.21 ^a^	0.14 ± 0.09 ^a^	0.22 ± 0.21 ^a^
**17**	Methyl isothiocyanate				0.0039											0.006
**18**	Tetramethylthiourea	0.0007	0.008 ± 0.007	0.0005	0.0041			0.031 ± 0.030 ^a^		0.011	0.0036 ± 0.0030 ^a^		0.0019 ± 0.0007 ^a^		0.0014 ± 0.0009 ^a^	0.009
**19**	Tetramethylurea	0.0020 ± 0.0016 ^a^	0.042 ± 0.035 ^a^	0.0035 ± 0.0020 ^a^	0.0046 ± 0.0040 ^a^		0.004	0.0057 ± 0.0050 ^a^	0.002 ± 0.001 ^a^	0.027	0.014 ± 0.007 ^a^	0.0032 ± 0.0020 ^a^	0.002 ± 0.001 ^a^	0.0031 ± 0.0004 ^a^	0.0040 ± 0.0022 ^a^	0.025
GDP pathway																
**20**	Camphene	0.0017 ± 0.0008 ^a^	0.0006± 0.0002 ^a^	0.0007 ± 0.0002 ^a^	0.0005 ± 0.0001 ^a^	0.0015 ± 0.0008 ^a^	0.0013 ± 0.0006 ^a^	0.0017 ± 0.0009 ^a^	0.00067 ± 0.00018 ^a^	0.014 ± 0.013 ^a^	0.00141± 0.00015 ^a^	0.00044 ± 0.00009 ^a^	0.00041 ± 0.00013 ^a^	0.00020 ± 0.00002 ^a^	0.00032 ± 0.00015 ^a^	0.00057 ± 0.00031 ^a^
**21**	3-Carene	0.013 ± 0.006 ^a^	0.014 ± 0.003 ^a^	0.012 ± 0.002 ^a^	0.009 ± 0.003 ^a^	0.019 ± 0.003 ^a^	0.005 ± 0.003 ^a^	0.014 ± 0.006 ^a^	0.014 ± 0.006 ^a^	0.0147 ± 0.0027 ^a^	0.025 ± 0.00 9 ^b^	0.004 ± 0.002 ^a^	0.0017 ± 0.0009 ^a^	0.0025 ± 0.0010 ^a^	0.0062 ± 0.0027 ^a^	0.0049 ± 0.0032 ^a^
**22**	Limonene	0.013 ± 0.008 ^a^	0.005 ± 0.001 ^a^	0.005 ± 0.001 ^a^	0.0019 ± 0.0006 ^a^	0.0040 ± 0.0005 ^a^	0.003	0.003 ± 0.002 ^a^	0.004 ± 0.002 ^a^	0.0021 ± 0.0004 ^a^	0.004 ± 0.002 ^a^	0.0023 ± 0.0016 ^a^	0.0006 ± 0.0001 ^a^	0.0005 ± 0.0002 ^a^	0.002 ± 0.001 ^a^	0.0013
**23**	α-Pinene	0.025 ± 0.006 ^a^	0.019 ± 0.004 ^a^	0.017 ± 0.003 ^a^	0.015 ± 0.003 ^b^	0.022 ± 0.004 ^a^	0.0080 ± 0.0001 ^a^	0.014 ± 0.006 ^a^	0.012 ± 0.007 ^a^	0.016 ± 0.005 ^a^	0.04 ± 0.01 ^a^	0.009 ± 0.004 ^a^	0.005 ± 0.001 ^a^	0.005 ± 0.003 ^a^	0.012 ± 0.002 ^a^	0.012 ± 0.007 ^a^
**24**	β-Pinene	0.0012 ± 0.0002 ^a^	0.0012± 0.0004 ^a^	0.0007 ± 0.0003 ^a^	0.0005 ± 0.0002 ^a^	0.0008 ± 0.0001 ^a^	0.0004	0.0005	0.002	0.00076 ± 0.00003 ^a^	0.0021 ± 0.0007 ^a^	0.0021 ± 0.0013 ^a^	0.00028 ± 0.00002 ^a^	0.00049 ± 0.00001 ^a^	0.0006 ± 0.0001 ^a^	0.0006 ± 0.0003 ^a^
GGDP pathway																
**25**	Geranyl acetone	0.0108 ± 0.0039 ^a^	0.036 ± 0.018 ^a^	0.0090 ± 0.0034 ^a^	0.013 ± 0.005 ^a^	0.015 ± 0.006 ^a^	0.030 ± 0.006 ^a^	0.042 ± 0.027 ^a^	0.049 ± 0.020 ^a^	0.058 ± 0.050 ^a^	0.049 ± 0.008 ^a^	0.010 ± 0.007 ^a^	0.0071 ± 0.0048 ^a^	0.0029 ± 0.0016 ^a^	0.013 ± 0.003 ^a^	0.0063 ± 0.0056 ^a^
**26**	6-Methyl-5-hepten-2-one	0.0034 ± 0.0015 ^a^	0.062 ± 0.047 ^a^	0.010 ± 0.005 ^a^	0.0077 ± 0.0031 ^a^	0.014 ± 0.008 ^a^	0.0031 ± 0.0015 ^a^	0.013 ± 0.010 ^a^	0.055 ± 0.027 ^a^	0.0077 ± 0.0050 ^a^	0.0057 ± 0.0014 ^a^	0.0087 ± 0.0081 ^a^	0.0042 ± 0.0033 ^a^	0.0037 ± 0.0022 ^a^	0.020 ± 0.008 ^a^	0.00033 ± 0.00011 ^a^
Saturated aldehydes																
**27**	Decanal	0.0132 ± 0.0030 ^a^	0.077 ± 0.030 ^b^	0.026 ± 0.007 ^a^	0.018 ± 0.006 ^a^	0.030 ± 0.006 ^a^	0.060 ± 0.044 ^a^	0.030 ± 0.016 ^a^	0.071 ± 0.012 ^a^	0.029 ± 0.016 ^a^	0.046 ± 0.017 ^a^	0.035 ± 0.016 ^a^	0.0190 ± 0.0070 ^a^	0.013 ± 0.002 ^a^	0.050 ± 0.027 ^a^	0.0029 ± 0.0008 ^a^
**28**	Heptanal	0.0072 ± 0.0011 ^a^	0.010 ± 0.003 ^b^	0.0042 ± 0.0010 ^a^	0.0053 ± 0.0011 ^a^	0.0071 ± 0.0020 ^a^	0.0055 ± 0.0020 ^a^	0.0040 ± 0.0003 ^a^	0.014 ± 0.005 ^a^	0.0072 ± 0.0020 ^a^	0.0078 ± 0.0002 ^a^	0.004 ± 0.001 ^a^	0.003 ± 0.001 ^a^	0.0019 ± 0.0003 ^a^	0.0083 ± 0.0006 ^b^	0.0014 ± 0.0001 ^a^
**29**	Nonanal	0.0182 ± 0.0043 ^a^	0.063 ± 0.020 ^b^	0.025 ± 0.007 ^a^	0.014 ± 0.005 ^a^	0.022 ± 0.007 ^a^	0.035 ± 0.020 ^a^	0.026 ± 0.008 ^a^	0.074 ± 0.020 ^a^	0.026 ± 0.009 ^a^	0.033 ± 0.018 ^a^	0.019 ± 0.007 ^a^	0.011 ± 0.004 ^a^	0.010 ± 0.002 ^a^	0.050 ± 0.017 ^a^	0.0026 ± 0.0003 ^a^
**30**	Octanal	0.0086 ± 0.0010 ^a^	0.022 ± 0.007 ^b^	0.011 ± 0.003 ^a^	0.006 ± 0.002 ^a^	0.012 ± 0.002 ^a^	0.013 ± 0.006 ^a^	0.0076 ± 0.0020 ^a^	0.033 ± 0.012 ^a^	0.0068 ± 0.0022 ^a^	0.0090 ± 0.0020 ^a^	0.012 ± 0.006 ^a^	0.004 ± 0.001 ^a^	0.0040 ± 0.0012 ^a^	0.021 ± 0.005 ^a^	0.0013 ± 0.0003 ^a^

The compounds were divided among key volatile groups: volatiles of the lipoxygenase (LOX) pathway, glucosinolate breakdown products, geranyl diphosphate (GDP) pathway volatiles, geranylgeranyl diphosphate (GGDP) pathway volatiles and saturated aldehydes. * Data without SE values correspond to measurements where the given compound was observed only in one plant among all the replicates. Emission rates between control and stressed plants at different time-points within the treatments were compared by paired sample *t*-tests and significant differences (*p* < 0.05) are indicated by lowercase letters.

## Data Availability

Data available in a publicly accessible repository after the manuscript is published.

## Sample Availability

Samples of the compounds are not available from the authors.

## References

[1] Lacour S.A., de Monte M., Diot P., Brocca J., Veron N., Colin P., Leblond V. (2006). Relationship between ozone and temperature during the 2003 heat wave in France: Consequences for health data analysis. BMC Public Health.

[2] Langematz U. (2019). Stratospheric ozone: Down and up through the anthropocene. Chemtexts.

[3] Ainsworth E.A. (2017). Understanding and improving global crop response to ozone pollution. Plant J..

[4] Bais A.F., Bernhard G., McKenzie R.L., Aucamp P.J., Young P.J., Ilyas M., Jockel P., Deushi M. (2019). Ozone-climate interactions and effects on solar ultraviolet radiation. Photochem. Photobiol. Sci..

[5] Chipperfield M.P., Bekki S., Dhomse S., Harris N.R.P., Hassler B., Hossaini R., Steinbrecht W., Thieblemont R., Weber M. (2017). Detecting recovery of the stratospheric ozone layer. Nature.

[6] Dhomse S.S., Feng W., Montzka S.A., Hossaini R., Keeble J., Pyle J.A., Daniel J.S., Chipperfield M.P. (2019). Delay in recovery of the Antarctic ozone hole from unexpected CFC-11 emissions. Nat. Commun..

[7] Feng Z.Z., Calatayud V., Zhu J.G., Kobayashi K. (2018). Ozone exposure- and flux-based response relationships with photosynthesis of winter wheat under fully open air condition. Sci. Total Environ..

[8] Masutomi Y., Kinose Y., Takimoto T., Yonekura T., Oue H., Kobayashi K. (2019). Ozone changes the linear relationship between photosynthesis and stomatal conductance and decreases water use efficiency in rice. Sci. Total Environ..

[9] Chaudhary N., Agrawal S.B. (2015). The role of elevated ozone on growth, yield and seed quality amongst six cultivars of mung bean. Ecotoxicol. Environ. Saf..

[10] Schaub M., Haeni M., Calatayud V., Ferretti M., Gottardini E. (2018). Forests Ozone Concentrations Are Decreasing but Exposure Remains High in European Forests.

[11] Querol X., Alastuey A., Gangoiti G., Perez N., Lee H.K., Eun H.R., Park Y., Mantilla E., Escudero M., Titos G. (2018). Phenomenology of summer ozone episodes over the Madrid Metropolitan Area, central Spain. Atmos. Chem. Phys..

[12] Archibald A.T., Turnock S.T., Griffiths P.T., Cox T., Derwent R.G., Knote C., Shin M. (2020). On the changes in surface ozone over the twenty-first century: Sensitivity to changes in surface temperature and chemical mechanisms. Philos. Trans. R. Soc. A Math. Phys. Eng. Sci..

[13] Chen C.P., Frank T.D., Long S.P. (2009). Is a short, sharp shock equivalent to long-term punishment? Contrasting the spatial pattern of acute and chronic ozone damage to soybean leaves via chlorophyll fluorescence imaging. Plant Cell Environ..

[14] Schraudner M., Moeder W., Wiese C., Van Camp W., Inze D., Langebartels C., Sandermann H. (1998). Ozone-induced oxidative burst in the ozone biomonitor plant, tobacco Bel W3. Plant J..

[15] Vollenweider P., Ottiger M., Gunthardt-Goerg M.S. (2003). Validation of leaf ozone symptoms in natural vegetation using microscopical methods. Environ. Pollut..

[16] Brosché M., Merilo E., Mayer F., Pechter P., Puzõrjova I., Brader G., Kangasjärvi J., Kollist H. (2010). Natural variation in ozone sensitivity among *Arabidopsis thaliana* accessions and its relation to stomatal conductance. Plant Cell Environ..

[17] Guidi L., Degl’Innocenti E., Genovesi S., Soldatini G.F. (2005). Photosynthetic process and activities of enzymes involved in the phenylpropanoid pathway in resistant and sensitive genotypes of *Lycopersicon esculentum* L. exposed to ozone. Plant Sci..

[18] Kangasjärvi J., Jaspers P., Kollist H. (2005). Signalling and cell death in ozone-exposed plants. Plant Cell Environ..

[19] Dickinson B.C., Chang C.J. (2011). Chemistry and biology of reactive oxygen species in signaling or stress responses. Nat. Chem. Biol..

[20] Beauchamp J., Wisthaler A., Hansel A., Kleist E., Miebach M., Niinemets Ü., Schurr U., Wildt J. (2005). Ozone induced emissions of biogenic VOC from tobacco: Relationships between ozone uptake and emission of LOX products. Plant Cell Environ..

[21] Rozpądek P., Nosek M., Ślesak I., Kunicki E., Dziurka M., Miszalski Z. (2015). Ozone fumigation increases the abundance of nutrients in Brassica vegetables: Broccoli (*Brassica oleracea* var. *italica*) and Chinese cabbage (*Brassica pekinensis*). Eur. Food Res. Technol..

[22] Rozpądek P., Ślesak I., Cebula S., Waligórski P., Dziurka M., Skoczowski A., Miszalski Z. (2013). Ozone fumigation results in accelerated growth and persistent changes in the antioxidant system of *Brassica oleracea* L. var. *capitata* f. *alba*. J. Plant Physiol..

[23] Li S., Harley P.C., Niinemets Ü. (2017). Ozone-induced foliar damage and release of stress volatiles is highly dependent on stomatal openness and priming by low-level ozone exposure in *Phaseolus vulgaris*. Plant Cell Environ..

[24] Martínez-Ballesta M.D., Moreno D.A., Carvajal M. (2013). The physiological importance of glucosinolates on plant response to abiotic stress in Brassica. Int. J. Mol. Sci..

[25] Dorokhov Y.L., Sheshukova E.V., Komarova T.V. (2018). Methanol in plant life. Front. Plant Sci..

[26] Peron A., Kaser L., Fitzky A.C., Graus M., Halbwirth H., Greiner J., Wohlfahrt G., Rewald B., Sanden H., Karl T. (2021). Combined effects of ozone and drought stress on the emission of biogenic volatile organic compounds from *Quercus robur* L.. Biogeosciences.

[27] Pokhrel Y., Felfelani F., Satoh Y., Boulange J., Burek P., Gadeke A., Gerten D., Gosling S.N., Grillakis M., Gudmundsson L. (2021). Global terrestrial water storage and drought severity under climate change. Nat. Clim. Chang..

[28] Taylor R.G., Scanlon B., Doll P., Rodell M., van Beek R., Wada Y., Longuevergne L., Leblanc M., Famiglietti J.S., Edmunds M. (2013). Ground water and climate change. Nat. Clim. Chang..

[29] Catola S., Centritto M., Cascone P., Ranieri A., Loreto F., Calamai L., Balestrini R., Guerrieri E. (2018). Effects of single or combined water deficit and aphid attack on tomato volatile organic compound (VOC) emission and plant-plant communication. Environ. Exp. Bot..

[30] Liu Y.L., Guo X.S., Ma M.S., Yu X.F. (2018). Maize seedlings response to drought stress and re-watering: Abscisic acid, a key regulator of physio-biochemical traits and gas exchange parameters. Pak. J. Bot..

[31] Saunier A., Ormeno E., Wortham H., Temime-Roussel B., Lecareux C., Boissard C., Fernandez C. (2017). Chronic drought decreases anabolic and catabolic BVOC emissions of *Quercus pubescens* in a Mediterranean forest. Front. Plant Sci..

[32] Niinemets Ü., Keenan T. (2014). Photosynthetic responses to stress in Mediterranean evergreens: Mechanisms and models. Environ. Exp. Bot..

[33] Jud W., Fischer L., Canaval E., Wohlfahrt G., Tissier A., Hansel A. (2016). Plant surface reactions: An opportunistic ozone defence mechanism impacting atmospheric chemistry. Atmos. Chem. Phys..

[34] Khan M.A.M., Ulrichs C., Mewis I. (2010). Influence of water stress on the glucosinolate profile of *Brassica oleracea* var. *italica* and the performance of *Brevicoryne brassicae* and *Myzus persicae*. Entomol. Exp. Appl..

[35] Salerno G., Frati F., Marino G., Ederli L., Pasqualini S., Loreto F., Colazza S., Centritto M. (2017). Effects of water stress on emission of volatile organic compounds by *Vicia faba*, and consequences for attraction of the egg parasitoid *Trissolcus basalis*. J. Pest Sci..

[36] Shang B., Yuan X.Y., Li P., Xu Y.S., Feng Z.Z. (2019). Effects of elevated ozone and water deficit on poplar saplings: Changes in carbon and nitrogen stocks and their allocation to different organs. For. Ecol. Manag..

[37] Kigathi R.N., Weisser W.W., Reichelt M., Gershenzon J., Unsicker S.B. (2019). Plant volatile emission depends on the species composition of the neighboring plant community. BMC Plant Biol..

[38] Kanagendran A., Pazouki L., Niinemets Ü. (2018). Differential regulation of volatile emission from *Eucalyptus globulus* leaves upon single and combined ozone and wounding treatments through recovery and relationships with ozone uptake. Environ. Exp. Bot..

[39] Niederbacher B., Winkler J.B., Schnitzler J.P. (2015). Volatile organic compounds as non-invasive markers for plant phenotyping. J. Exp. Bot..

[40] Niinemets Ü. (2010). Mild versus severe stress and BVOCs: Thresholds, priming and consequences. Trends Plant Sci..

[41] Jud W., Vanzo E., Li Z.R., Ghirardo A., Zimmer I., Sharkey T.D., Hansel A., Schnitzler J.P. (2016). Effects of heat and drought stress on post-illumination bursts of volatile organic compounds in isoprene-emitting and non-emitting poplar. Plant Cell Environ..

[42] Zhang J., Gao F., Jia H.X., Hu J.J., Feng Z.Z. (2019). Molecular response of poplar to single and combined ozone and drought. Sci. Total Environ..

[43] Buckley T.N. (2019). How do stomata respond to water status?. New Phytol..

[44] Hoshika Y., Omasa K., Paoletti E. (2013). Both ozone exposure and soil water stress are able to induce stomatal sluggishness. Environ. Exp. Bot..

[45] Otu-Larbi F., Conte A., Fares S., Wild O., Ashworth K. (2020). Current and future impacts of drought and ozone stress on Northern Hemisphere forests. Glob. Chang. Biol..

[46] Pääkkönen E., Vahala J., Pohjolai M., Holopainen T., Kärenlampi L. (1998). Physiological, stomatal and ultrastructural ozone responses in birch (*Betula pendula* Roth.) are modified by water stress. Plant Cell Environ..

[47] Kask K., Kännaste A., Talts E., Copolovici L., Niinemets Ü. (2016). How specialized volatiles respond to chronic and short-term physiological and shock heat stress in *Brassica nigra*. Plant Cell Environ..

[48] Veromann E., Toome M., Kännaste A., Kaasik R., Copolovici L., Flink J., Kovacs G., Narits L., Luik A., Niinemets Ü. (2013). Effects of nitrogen fertilization on insect pests, their parasitoids, plant diseases and volatile organic compounds in *Brassica napus*. Crop Prot..

[49] Kissen R., Rossiter J.T., Bones A.M. (2009). The ‘mustard oil bomb’: Not so easy to assemble?! Localization, expression and distribution of the components of the myrosinase enzyme system. Phytochem. Rev..

[50] Pang Q.Y., Chen S.X., Li L.X., Yan X.F. (2009). Characterization of glucosinolate-myrosinase system in developing salt cress *Thellungiella halophila*. Physiol. Plant..

[51] Mithen R.F. (2001). Glucosinolates and their degradation products. Adv. Bot. Res..

[52] Ponzio C., Papazian S., Albrectsen B.R., Dicke M., Gols R. (2017). Dual herbivore attack and herbivore density affect metabolic profiles of *Brassica nigra* leaves. Plant Cell Environ..

[53] Stam J.M., Chretien L., Dicke M., Poelman E.H. (2017). Response of *Brassica oleracea* to temporal variation in attack by two herbivores affects preference and performance of a third herbivore. Ecol. Entomol..

[54] Han Y.J., Gharibeshghi A., Mewis I., Forster N., Beck W., Ulrichs C. (2021). Effect of different durations of moderate ozone exposure on secondary metabolites of *Brassica campestris* L. ssp. *chinensis*. J. Hortic. Sci. Biotechnol..

[55] Himanen S.J., Nissinen A., Auriola S., Poppy G.M., Stewart C.N., Holopainen J.K., Nerg A.-M. (2008). Constitutive and herbivore-inducible glucosinolate concentrations in oilseed rape (*Brassica napus*) leaves are not affected by Bt Cry1Ac insertion but change under elevated atmospheric CO_2_ and O_3_. Planta.

[56] Khaling E., Li T., Holopainen J.K., Blande J.D. (2016). Elevated ozone modulates herbivore-induced volatile emissions of *Brassica nigra* and alters a tritrophic interaction. J. Chem. Ecol..

[57] Saunier A., Blande J.D. (2019). The effect of elevated ozone on floral chemistry of Brassicaceae species. Environ. Pollut..

[58] Brosset A., Saunier A., Kivimäenpää M., Blande J.D. (2020). Does ozone exposure affect herbivore-induced plant volatile emissions differently in wild and cultivated plants?. Environ. Sci. Pollut. Res..

[59] Dicke M., Baldwin I.T. (2010). The evolutionary context for herbivore-induced plant volatiles: Beyond the ‘cry for help’. Trends Plant Sci..

[60] Kessler A., Halitschke R., Poveda K. (2011). Herbivory-mediated pollinator limitation: Negative impacts of induced volatiles on plant-pollinator interactions. Ecology.

[61] Fatouros N.E., Lucas-Barbosa D., Weldegergis B.T., Pashalidou F.G., van Loon J.J.A., Dicke M., Harvey J.A., Gols R., Huigens M.E. (2012). Plant volatiles induced by herbivore egg deposition affect insects of different trophic levels. PLoS ONE.

[62] Jeschke V., Kearney E.E., Schramm K., Kunert G., Shekhov A., Gershenzon J., Vassao D.G. (2017). How glucosinolates affect generalist lepidopteran larvae: Growth, development and glucosinolate metabolism. Front. Plant Sci..

[63] Giron-Calva P.S., Li T., Blande J.D. (2016). Plant-plant interactions affect the susceptibility of plants to oviposition by pests but are disrupted by ozone pollution. Agric. Ecosyst. Environ..

[64] Augustine R., Bisht N.C. (2017). Regulation of glucosinolate metabolism: From model plant *Arabidopsis thaliana* to Brassica crops. Glucosinolates.

[65] Bonnet C., Lassueur S., Ponzio C., Gols R., Dicke M., Reymond P. (2017). Combined biotic stresses trigger similar transcriptomic responses but contrasting resistance against a chewing herbivore in *Brassica nigra*. BMC Plant Biol..

[66] Pineda A., Soler R., Pastor V., Li Y.H., Dicke M. (2017). Plant-mediated species networks: The modulating role of herbivore density. Ecol. Entomol..

[67] Douma J.C., Vermeulen P.J., Poelman E.H., Dicke M., Anten N.P.R. (2017). When does it pay off to prime for defense? A modeling analysis. New Phytol..

[68] Papazian S., Khaling E., Bonnet C., Lassueur S., Reymond P., Moritz T., Blande J.D., Albrectsen B.R. (2016). Central metabolic responses to ozone and herbivory affect photosynthesis and stomatal closure. Plant Physiol..

[69] Hetherington A.M., Woodward F.I. (2003). The role of stomata in sensing and driving environmental change. Nature.

[70] Vahisalu T., Puzõrjova I., Brosché M., Valk E., Lepiku M., Moldau H., Pechter P., Wang Y.S., Lindgren O., Salojärvi J. (2010). Ozone-triggered rapid stomatal response involves the production of reactive oxygen species, and is controlled by SLAC1 and OST1. Plant J..

[71] Niinemets Ü., Reichstein M. (2003). Controls on the emission of plant volatiles through stomata: Differential sensitivity of emission rates to stomatal closure explained. J. Geophys. Res. Atmos..

[72] Flexas J., Bota J., Escalona J.M., Sampol B., Medrano H. (2002). Effects of drought on photosynthesis in grapevines under field conditions: An evaluation of stomatal and mesophyll limitations. Funct. Plant Biol..

[73] Chaves M.M., Flexas J., Pinheiro C. (2009). Photosynthesis under drought and salt stress: Regulation mechanisms from whole plant to cell. Ann. Bot..

[74] Correia B., Hancock R.D., Amaral J., Gomez-Cadenas A., Valledor L., Pinto G. (2018). Combined drought and heat activates protective responses in *Eucalyptus globulus* that are not activated when subjected to drought or heat stress alone. Front. Plant Sci..

[75] Moldau H., Sõber J., Sõber A. (1993). Impact of acute ozone exposure on CO_2_ uptake by two cultivars of *Phaseolus vulgaris*. Photosynthetica.

[76] Kull O., Moldau H. (1994). Absorption of ozone on *Betula pendula* Roth leaf surface. Water Air Soil Pollut..

[77] Moldau H., Bichele I. (2002). Plasmalemma protection by the apoplast as assessed from above-zero ozone concentrations in leaf intercellular air spaces. Planta.

[78] Hassan I.A., Ashmore M.R., Bell J.N.B. (1994). Effects of O_3_ on the stomatal behaviour of Egyptian varieties of radish (*Raphanus sativus* L. cv. Baladey) and turnip (*Brassica rapa* L. cv. Sultani). New Phytol..

[79] Bergmann E., Bender J., Weigel H.J. (2017). Impact of tropospheric ozone on terrestrial biodiversity: A literature analysis to identify ozone sensitive taxa. J. Appl. Bot. Food Qual..

[80] Tardieu F., Parent B., Simonneau T. (2010). Control of leaf growth by abscisic acid: Hydraulic or non-hydraulic processes?. Plant Cell Environ..

[81] Wilkinson S., Davies W.J. (2009). Ozone suppresses soil drying- and abscisic acid (ABA)-induced stomatal closure via an ethylene-dependent mechanism. Plant Cell Environ..

[82] Hoshika Y., Katata G., Deushi M., Watanabe M., Koike T., Paoletti E. (2015). Ozone-induced stomatal sluggishness changes carbon and water balance of temperate deciduous forests. Sci. Rep..

[83] Alonso R., Elvira S., González-Fernández I., Calvete H., García-Gómez H., Bermejo V. (2014). Drought stress does not protect *Quercus ilex* L. from ozone effects: Results from a comparative study of two subspecies differing in ozone sensitivity. Plant Biol..

[84] Tingey D.T., Hogsett W.E. (1985). Water stress reduces ozone injury via a stomatal mechanism. Plant Physiol..

[85] Mittler R. (2006). Abiotic stress, the field environment and stress combination. Trends Plant Sci..

[86] Sconiers W.B., Rowland D.L., Eubanks M.D. (2020). Pulsed drought: The effects of varying water stress on plant physiology and predicting herbivore response. Crop Sci..

[87] Guidi L., Tonini M., Soldatini G.F. (2000). Effects of high light and ozone fumigation on photosynthesis in *Phaseolus vulgaris*. Plant Physiol. Biochem..

[88] Lombardozzi D., Sparks J.P., Bonan G., Levis S. (2012). Ozone exposure causes a decoupling of conductance and photosynthesis: Implications for the Ball-Berry stomatal conductance model. Oecologia.

[89] Kanagendran A., Pazouki L., Li S., Liu B., Kännaste A., Niinemets Ü. (2018). Ozone-triggered surface uptake and stress volatile emissions in *Nicotiana tabacum* ‘Wisconsin’. J. Exp. Bot..

[90] Zhang W.W., Feng Z.Z., Wang X.K., Niu J.F. (2014). Impacts of elevated ozone on growth and photosynthesis of *Metasequoia glyptostroboides* Hu et Cheng. Plant Sci..

[91] Singh E., Tiwari S., Agrawal M. (2009). Effects of elevated ozone on photosynthesis and stomatal conductance of two soybean varieties: A case study to assess impacts of one component of predicted global climate change. Plant Biol..

[92] Paoletti E., Grulke N.E. (2005). Does living in elevated CO_2_ ameliorate tree response to ozone? A review on stomatal responses. Environ. Pollut..

[93] Fahad S., Bajwa A.A., Nazir U., Anjum S.A., Farooq A., Zohaib A., Sadia S., Nasim W., Adkins S., Saud S. (2017). Crop production under drought and heat stress: Plant responses and management options. Front. Plant Sci..

[94] Osakabe Y., Osakabe K., Shinozaki K., Tran L.S.P. (2014). Response of plants to water stress. Front. Plant Sci..

[95] Sun Z.H., Shen Y., Niinemets Ü. (2020). Responses of isoprene emission and photochemical efficiency to severe drought combined with prolonged hot weather in hybrid Populus. J. Exp. Bot..

[96] Mewis I., Khan M.A.M., Glawischnig E., Schreiner M., Ulrichs C. (2012). Water stress and aphid feeding differentially influence metabolite composition in *Arabidopsis thaliana* (L.). PLoS ONE.

[97] Weldegergis B.T., Zhu F., Poelman E.H., Dicke M. (2018). Drought stress affects plant metabolites and herbivore preference but not host location by its parasitoids. Oecologia.

[98] Niinemets Ü., Arneth A., Kuhn U., Monson R.K., Peñuelas J., Staudt M. (2010). The emission factor of volatile isoprenoids: Stress, acclimation, and developmental responses. Biogeosciences.

[99] Khaling E., Papazian S., Poelman E.H., Holopainen J.K., Albrectsen B.R., Blande J.D. (2015). Ozone affects growth and development of *Pieris brassicae* on the wild host plant *Brassica nigra*. Environ. Pollut..

[100] Bailey A., Burkey K., Taggart M., Rufty T. (2019). Leaf traits that contribute to differential ozone response in ozone-tolerant and sensitive soybean genotypes. Plants.

[101] Li S., Tosens T., Harley P.C., Jiang Y.F., Kanagendran A., Grosberg M., Jaamets K., Niinemets Ü. (2018). Glandular trichomes as a barrier against atmospheric oxidative stress: Relationships with ozone uptake, leaf damage, and emission of LOX products across a diverse set of species. Plant Cell Environ..

[102] Turlings T.C.J., Loughrin J.H., McCall P.J., Rose U.S.R., Lewis W.J., Tumlinson J.H. (1995). How caterpillar-damaged plants protect themselves by attracting parasitic wasps. Proc. Natl. Acad. Sci. USA.

[103] Smith L., Beck J.J. (2015). Duration of emission of volatile organic compounds from mechanically damaged plant leaves. J. Plant Physiol..

[104] Turan S., Kask K., Kanagendran A., Li S., Anni R., Talts E., Rasulov B., Kännaste A., Niinemets Ü. (2019). Lethal heat stress-dependent volatile emissions from tobacco leaves: What happens beyond the thermal edge?. J. Exp. Bot..

[105] Portillo-Estrada M., Kazantsev T., Talts E., Tosens T., Niinemets Ü. (2015). Emission timetable and quantitative patterns of wound-induced volatiles across different leaf damage treatments in aspen (*Populus tremula*). J. Chem. Ecol..

[106] Brilli F., Ruuskanen T.M., Schnitzhofer R., Muller M., Breitenlechner M., Bittner V., Wohlfahrt G., Loreto F., Hansel A. (2011). Detection of plant volatiles after leaf wounding and darkening by proton transfer reaction “time-of-flight” mass spectrometry (PTR-TOF). PLoS ONE.

[107] Jiang Y.F., Ye J.Y., Li S., Niinemets Ü. (2017). Methyl jasmonate-induced emission of biogenic volatiles is biphasic in cucumber: A high-resolution analysis of dose dependence. J. Exp. Bot..

[108] Hu Z.-H., Leng P.-S., Shen Y.-B., Wang W.-H. (2011). Emissions of saturated C_6_-C_10_ aldehydes from poplar (*Populus simonii* × *P. pyramidalis* ‘Opera 8277’) cuttings at different levels of light intensity. J. For. Res..

[109] Shen J., Tieman D., Jones J.B., Taylor M.G., Schmelz E., Huffaker A., Bies D., Chen K., Klee H.J. (2014). A 13-lipoxygenase, TomloxC, is essential for synthesis of C_5_ flavour volatiles in tomato. J. Exp. Bot..

[110] Heiden A.C., Kobel K., Langebartels C., Schuh-Thomas G., Wildt J. (2003). Emissions of oxygenated volatile organic compounds from plants—Part I: Emissions from lipoxygenase activity. J. Atmos. Chem..

[111] Adams A., Bouckaert C., Van Lancker F., De Meulenaer B., De Kimpe N. (2011). Amino acid catalysis of 2-alkylfuran formation from lipid oxidation-derived α,β-unsaturated aldehydes. J. Agric. Food Chem..

[112] Redovniković I.R., Glivetić T., Delonga K., Vorkapić-Furač J. (2008). Glucosinolates and their potential role in plant. Period. Biol..

[113] Miękus N., Marszałek K., Podlacha M., Iqbal A., Puchalski C., Świergiel A.H. (2020). Health benefits of plant-derived sulfur compounds, glucosinolates, and organosulfur compounds. Molecules.

[114] Tulio A.Z., Yamanaka H., Ueda Y., Imahori Y. (2002). Formation of methanethiol and dimethyl disulfide in crushed tissues of broccoli florets and their inhibition by freeze-thawing. J. Agric. Food Chem..

[115] Dan K., Nagata M., Yamashita I. (1999). Mechanism of off-flavor production in Brassica vegetables under anaerobic conditions. JARQ-Jpn. Agric. Res. Q..

[116] Ratzka A., Vogel H., Kliebenstein D.J., Mitchell-Olds T., Kroymann J. (2002). Disarming the mustard oil bomb. Proc. Natl. Acad. Sci. USA.

[117] Maffei M.E. (2010). Sites of synthesis, biochemistry and functional role of plant volatiles. S. Afr. J. Bot..

[118] Staudt M., Lhoutellier L. (2011). Monoterpene and sesquiterpene emissions from *Quercus coccifera* exhibit interacting responses to light and temperature. Biogeosciences.

[119] Loreto F., Pinelli P., Manes F., Kollist H. (2004). Impact of ozone on monoterpene emissions and evidence for an isoprene-like antioxidant action of monoterpenes emitted by *Quercus ilex* leaves. Tree Physiol..

[120] Loreto F., Förster A., Durr M., Csiky O., Seufert G. (1998). On the monoterpene emission under heat stress and on the increased thermotolerance of leaves of *Quercus ilex* L. fumigated with selected monoterpenes. Plant Cell Environ..

[121] Acton W.J.F., Jud W., Ghirardo A., Wohlfahrt G., Hewitt C.N., Taylor J.E., Hansel A. (2018). The effect of ozone fumigation on the biogenic volatile organic compounds (BVOCs) emitted from *Brassica napus* above- and below-ground. PLoS ONE.

[122] Cui H.Y., Wei J.I., Su J.W., Li C.Y., Ge F. (2016). Elevated O_3_ increases volatile organic compounds via jasmonic acid pathway that promote the preference of parasitoid *Encarsia formosa* for tomato plants. Plant Sci..

[123] Nogués I., Brilli F., Loreto F. (2006). Dimethylallyl diphosphate and geranyl diphosphate pools of plant species characterized by different isoprenoid emissions. Plant Physiol..

[124] Fruekilde P., Hjorth J., Jensen N.R., Kotzias D., Larsen B. (1998). Ozonolysis at vegetation surfaces: A source of acetone, 4-oxopentanal, 6-methyl-5-hepten-2-one, and geranyl acetone in the troposphere. Atmos. Environ..

[125] Pellegrini E., Cioni P.L., Francini A., Lorenzini G., Nali C., Flamini G. (2012). Volatiles emission patterns in Poplar clones clones varying in response to ozone. J. Chem. Ecol..

[126] Simkin A.J., Schwartz S.H., Auldridge M., Taylor M.G., Klee H.J. (2004). The tomato carotenoid cleavage dioxygenase 1 genes contribute to the formation of the flavor volatiles beta-ionone, pseudoionone, and geranylacetone. Plant J..

[127] Walter M.H., Strack D. (2011). Carotenoids and their cleavage products: Biosynthesis and functions. Nat. Prod. Rep..

[128] Beisel K.G., Jahnke S., Hofmann D., Koppchen S., Schurr U., Matsubara S. (2010). Continuous turnover of carotenes and chlorophyll a in mature leaves of Arabidopsis revealed by ^14^CO_2_ pulse-chase labeling. Plant Physiol..

[129] Tieman D.M., Zeigler M., Schmelz E.A., Taylor M.G., Bliss P., Kirst M., Klee H.J. (2006). Identification of loci affecting flavour volatile emissions in tomato fruits. J. Exp. Bot..

[130] García-Plazaola J.I., Portillo-Estrada M., Fernandez-Marin B., Kännaste A., Niinemets Ü. (2017). Emissions of carotenoid cleavage products upon heat shock and mechanical wounding from a foliose lichen. Environ. Exp. Bot..

[131] Desikan R., Mackerness S.A.H., Hancock J.T., Neill S.J. (2001). Regulation of the Arabidopsis transcriptome by oxidative stress. Plant Physiol..

[132] Kaiser H. (2009). The relation between stomatal aperture and gas exchange under consideration of pore geometry and diffusional resistance in the mesophyll. Plant Cell Environ..

[133] Mano J., Biswas M.S., Sugimoto K. (2019). Reactive carbonyl species: A missing link in ROS signaling. Plants.

[134] Daszkowska-Golec A., Szarejko I. (2013). Open or close the gate—Stomata action under the control of phytohormones in drought stress conditions. Front. Plant Sci..

[135] Bruinsma M., Lucas-Barbosa D., ten Broeke C.J.M., van Dam N.M., van Beek T.A., Dicke M., van Loon J.J.A. (2014). Folivory affects composition of nectar, floral odor and modifies pollinator behavior. J. Chem. Ecol..

[136] Wildt J., Kobel K., Schuh-Thomas G., Heiden A.C. (2003). Emissions of oxygenated volatile organic compounds from plants—Part II: Emissions of saturated aldehydes. J. Atmos. Chem..

[137] Giron-Calva P.S., Li T., Blande J.D. (2017). Volatile-mediated interactions between cabbage plants in the field and the impact of ozone pollution. J. Chem. Ecol..

[138] Copolovici L., Niinemets Ü. (2010). Flooding induced emissions of volatile signalling compounds in three tree species with differing waterlogging tolerance. Plant Cell Environ..

[139] Von Caemmerer S., Farquhar G.D. (1981). Some relationships between the biochemistry of photosynthesis and the gas exchange of leaves. Planta.

[140] Jardine K.J., Monson R.K., Abrell L., Saleska S.R., Arneth A., Jardine A., Ishida F.Y., Yanez Serrano A.M., Artaxo P., Karl T. (2012). Within-plant isoprene oxidation confirmed by direct emissions of oxidation products methyl vinyl ketone and methacrolein. Glob. Chang. Biol..

[141] Kännaste A., Copolovici L., Niinemets Ü., Rodriguez Concepcion M. (2014). Gas chromatography-mass spectrometry method for determination of biogenic volatile organic compounds emitted by plants. Plant Isoprenoids: Methods and Protocols.

[142] Niinemets Ü., Kuhn U., Harley P.C., Staudt M., Arneth A., Cescatti A., Ciccioli P., Copolovici L., Geron C., Guenther A. (2011). Estimations of isoprenoid emission capacity from enclosure studies: Measurements, data processing, quality and standardized measurement protocols. Biogeosciences.

[143] Wold S., Esbensen K., Geladi P. (1987). Principal component analysis. Chemom. Intell. Lab. Syst..

[144] Niinemets Ü. (2020). Leaf trait plasticity and evolution in different plant functional types. Annu. Plant Rev..

[145] Guidi L., Nali C., Lorenzini G., Filippi F., Soldatini G.F. (2001). Effect of chronic ozone fumigation on the photosynthetic process of poplar clones showing different sensitivity. Environ. Pollut..

[146] Mills G., Hayes F., Wilkinson S., Davies W.J. (2009). Chronic exposure to increasing background ozone impairs stomatal functioning in grassland species. Glob. Chang. Biol..

